# Recognition of two distinct elements in the RNA substrate by the RNA-binding domain of the *T. thermophilus* DEAD box helicase Hera

**DOI:** 10.1093/nar/gkt323

**Published:** 2013-04-25

**Authors:** Lenz Steimer, Jan Philip Wurm, Martin H. Linden, Markus G. Rudolph, Jens Wöhnert, Dagmar Klostermeier

**Affiliations:** ^1^Institute for Physical Chemistry, University of Muenster, Corrensstrasse 30, D-48149 Muenster, Germany, ^2^Center for Biomolecular Magnetic Resonance (BMRZ), Institute for Molecular Biosciences, University of Frankfurt, Max-von-Laue-Strasse 9, D-60438 Frankfurt/M., Germany, ^3^University of Basel, Biozentrum, Klingelbergstrasse 70, CH-4056 Basel, Switzerland and ^4^F. Hoffmann-La Roche AG, pRED, Pharma Research & Early Development, Discovery Technologies, Grenzacherstrasse 124, CH-4070 Basel, Switzerland

## Abstract

DEAD box helicases catalyze the ATP-dependent destabilization of RNA duplexes. Whereas duplex separation is mediated by the helicase core shared by all members of the family, flanking domains often contribute to binding of the RNA substrate. The Thermus thermophilus DEAD-box helicase Hera (for “heat-resistant RNA-binding ATPase”) contains a C-terminal RNA-binding domain (RBD). We have analyzed RNA binding to the Hera RBD by a combination of mutational analyses, nuclear magnetic resonance and X-ray crystallography, and identify residues on helix α1 and the C-terminus as the main determinants for high-affinity RNA binding. A crystal structure of the RBD in complex with a single-stranded RNA resolves the RNA–protein interactions in the RBD core region around helix α1. Differences in RNA binding to the Hera RBD and to the structurally similar RBD of the *Bacillus subtilis* DEAD box helicase YxiN illustrate the versatility of RNA recognition motifs as RNA-binding platforms. Comparison of chemical shift perturbation patterns elicited by different RNAs, and the effect of sequence changes in the RNA on binding and unwinding show that the RBD binds a single-stranded RNA region at the core and simultaneously contacts double-stranded RNA through its C-terminal tail. The helicase core then unwinds an adjacent RNA duplex. Overall, the mode of RNA binding by Hera is consistent with a possible function as a general RNA chaperone.

## INTRODUCTION

DEAD box helicases unwind RNA duplexes in an adenosine triphosphate (ATP)-dependent reaction [reviewed in (1,2)]. Members of the DEAD box family share a common helicase core of two flexibly linked globular domains that carry the helicase signature motifs mediating ATP binding and hydrolysis, RNA binding and duplex unwinding. Many DEAD box helicases comprise additional domains flanking the core region that affect nucleotide binding and hydrolysis, and contribute to RNA binding and specificity, or binding of protein partners (3–12), or to duplex destabilization (13).

**Figure 1.**
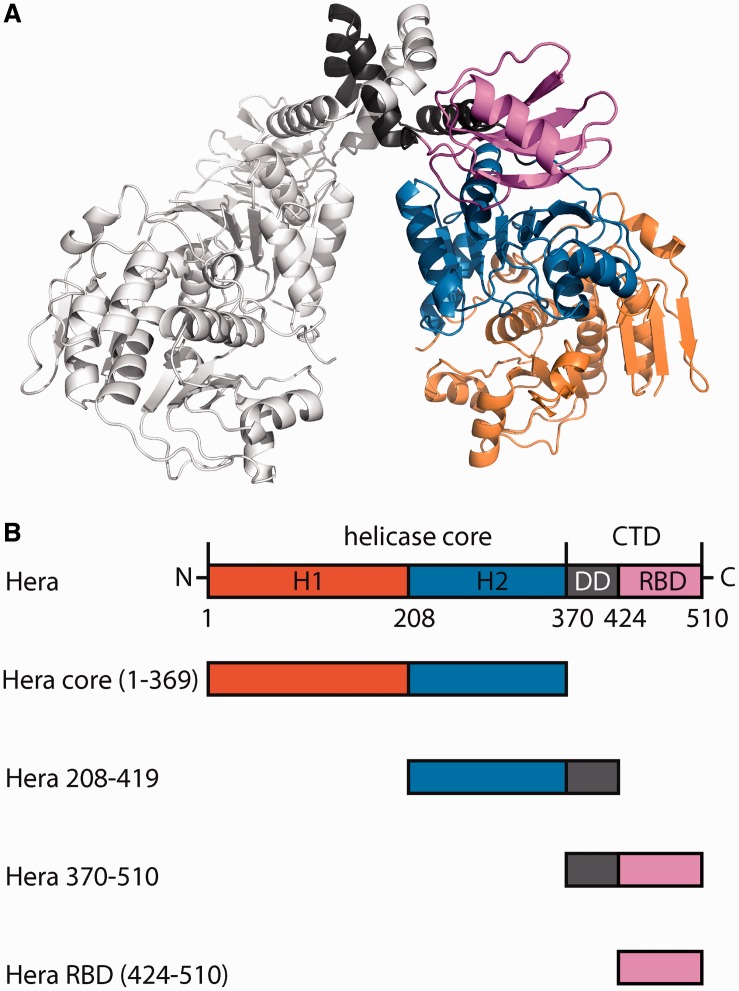
Architecture of Hera and constructs used in this study. (**A**) Structural model of the Hera dimer (15,17,18). One protomer is depicted in gray. For the second protomer, the N-terminal and C-terminal RecA domains (RecA_N, RecA_C) of the helicase core are shown in orange and blue, respectively, the dimerization domain (DD) in gray and the RNA-binding domain (RBD) in magenta. (**B**) Domain structure of Hera and deletion constructs used in this study.

The ‘heat-resistant RNA-dependent ATPase’ Hera is a DEAD box protein from *Thermus thermophilus* (14). Hera consists of a helicase core, followed by a bipartite C-terminal extension that contains a dimerization domain (DD) and an RNA-binding domain (RBD) (Figure 1A) (15–18). The DD mediates the formation of a stable dimer (17), even at picomolar concentrations [(7), Linden & Klostermeier, unpublished]. The RBD is responsible for binding of Hera to 23S rRNA fragments comprising hairpin 92 and for binding of RNaseP RNA (7). Both RNAs induce a conformational change in the Hera helicase core (7) to a compact closed state, in which the two domains of the helicase core form the catalytic site for ATP hydrolysis and the RNA-binding site (19). Hera unwinds a minimal RNA substrate comprising hairpin 92 and the adjacent helix 91 of the 23S rRNA in an ATP-dependent reaction (7). However, the *in vivo* role of Hera is currently unclear.

The Hera RBD forms a modified RNA recognition motif (RRM) (15). The first 12 amino acids fold into a short double-β-hairpin structure that attaches the RBD to the C-terminal RecA domain of the helicase core (15). The RRM itself consists of a central four-stranded β-sheet, flanked by an α-helix, and a long loop connecting β-strands 3 and 4 that replaces helix α2 present in canonical RRMs. The C-terminal 10 amino acids are disordered, and the last five residues in the structure are distended from the main body of the RRM (15). Classical RRMs share two conserved regions on the central β-sheet, termed RNP1 and RNP2. In the conventional RNA-binding mode, RNA is bound across the central β-sheet, involving conserved aromatic residues in RNP1 and RNP2 (e.g. U1A (20)). However, in the Hera RBD, these sequences deviate significantly from the consensus, implying a non-classical RNA-binding mode. In alternative binding modes, RNAs can contact the central β-sheet, plus the loop connecting β2 and β3, resulting in RNA binding perpendicular to the strands of the β-sheet (e.g. SRp20, (21)). So-called quasi-RRMs (qRRMs) have been shown to bind RNA through loops, without contributions from the β-sheets (22). However, intermediate forms between these classes were recently found that suggest a continuum of RNA-binding modes, indicating that RRMs are versatile RNA-binding platforms (recently reviewed in (23)).

We have dissected the binding of RNA to the Hera RBD by a combination of mutational analyses, nuclear magnetic resonance (NMR) and X-ray crystallography. Fluorescence anisotropy titrations and NMR chemical shift perturbation (CSP) experiments identify residues on α-helix α1 and the C-terminal tail as the main determinants for high-affinity RNA binding to the Hera RBD. Despite its important role in RNA binding, the C-terminal tail does not adopt a regular secondary structure in the presence of RNA, although its flexibility is reduced. A crystal structure of the RBD in complex with a tetranucleotide RNA resolves the RNA-protein interactions in the central region of the binding site, and illustrates major differences to RNA binding by the RBD of the *Bacillus subtilis* DEAD box helicase YxiN (24). Comparison of the CSP patterns elicited by different RNAs, and of their binding and unwinding, points towards an interaction of Hera_RBD with single-stranded RNA through the RBD core region, and with double-stranded RNA through the C-terminal tail. The helicase core then unwinds adjacent double-stranded regions.

## MATERIALS AND METHODS

### Protein production and purification

Hera and deletion constructs Hera_1-369 (Hera_core), Hera_208-419 (Hera_RecA_C_DD) and Hera 370-510 (Hera_DD_RBD, Figure 1B) were produced in *Escherichia coli* Rosetta(DE3) in autoinducing medium (25) and purified as previously described (7,17). The glutathione-S-transferase (GST)_Hera_RBD fusion protein was purified on glutathione sepharose, Ni^2+^-nitrilotriacetate (NTA) sepharose and an S75 size-exclusion column, as described (15,16). The ^15^N- and ^15^N,^13^C-labeled Hera_RBD was produced as an N-terminal GST-fusion in M9 minimal medium (modified after (26)), supplemented with 1 g/l ^15^NH_4_Cl and/or 2 g/l ^13^C-glucose, respectively, and purified as the unlabeled protein.

### RNA substrates

RNA oligonucleotides were from Purimex or Dharmacon. The sequences were: 5′-GCAGGUCCCAAGGGU UGGGC UGUUC GCCCAUU-3′ (32mer), 5′-CGAGGUCCCAAGGGU UGGGC UGUU GCCCAUU-3′ (32mer_loop4), 5′-CGAGGUCCCAAGGGU UGGGC UGU*U*UC GCCCAUU-3′ (32mer_loop6), 5′-CGAGGUCCCAAGGGU UGGGC U*U*U*A*C GCCCAUU-3′ (32mer_loopmut), 5′-CGAGGUCCCAAGGGU UGGC UGUUC GCCAUU-3′ (32mer_stem4), 5′-CGAGGUCCCAAGGGU UAGGGC UGUUC GCCCUAUU-3′ (32mer_stem6), 5′-CGAGGUCCCAAGGGU UGGG*A* UGUUC *U*CCUAUU-3 (32mer_stemmut), 5′-UUGGGACCU-3′ (9mer), 5′-GGGUUGGGCUGUUCGCCCAUU-3′ (21mer), 5′-CACUUGGGCUGUUCGCCCAUU-3′ (21mer_mod), 5′-GGGUUGGGCUAUUCGCCCAUU-3′ (21mer_modloop), 5′-GUU**GGGC**UGUUCGCCCAUU-3′ (19mer), 5′-GGGCC-3 (5mer) and 5′-GGGC-3′ (4mer). The regions forming the stem of hairpin 92 are underlined. Sequence variations are indicated in italics. The four nucleotides of the 19mer that were observed in the crystal structure are highlighted in bold.

G,U-^15^N-labeled 21mer was generated by *in vitro* transcription from a linearized plasmid using T7 polymerase and ^15^N-labeled GTP and UTP (Silantes GmbH). Linearization was performed by cleavage at an XbaI-restriction site inserted after the region coding for the 21mer, resulting in four additional nucleotides (CUAG) at the 3′-end. The transcript was purified by denaturing polyacrylamide gel electrophoresis (PAGE), heated to 95°C for 5 min and folded by 5-fold dilution into ice-cold H_2_O, as described previously (27).

### Determination of K_d_ values in fluorescence equilibrium titrations

K_d_ values for Hera/RNA complexes were determined in fluorescence anisotropy titrations of 50 nM RNA (4mer or 32mer), 5′-labeled with fluorescein, in 50 mM Tris/HCl, pH 7.5, 150 mM NaCl with Hera, Hera_core or GST-Hera_RBD at 25°C, as described (7), using a Jobin Yvon FluoroMax3 fluorimeter. Fluorescence was excited at 495 nm (5 nm bandwidth) and detected at 530 nm (10 nm bandwidth). The equilibration time was 2 min, which is sufficient to allow for complex formation (Supplementary Figure S1). Data were analyzed using the solution of the quadratic equation that describes a 1:1 complex formation (eq. 1)
(1)
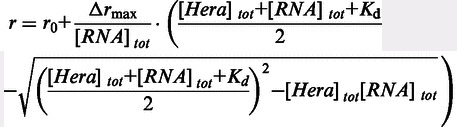

where r_0_ is the anisotropy of free RNA, Δr_max_ is the amplitude, [Hera]_tot_ is the total Hera concentration and [RNA]_tot_ is the total RNA concentration.

For stoichiometic titrations, 5 µM of 5′-fluorescein-labeled 32mer were titrated with Hera and GST-Hera_RBD until saturation was achieved.

### Electrophoretic mobility shift assays

To determine K_d_ values by electrophoretic shift assays, 6 µM of 5′-fluorescein-labeled substrate RNA (32mer or 21mer) were mixed with increasing concentrations of GST-Hera_RBD in 50 mM Tris/HCl, pH 7.5, 150 mM NaCl and 12% (v/v) glycerol, incubated at 25°C for 30 min and subjected to electrophoresis on a 15% (32mer) or 18% (21mer) polyacrylamide gel. Gel electrophoresis was performed at 140 V for 30 min in 1 × TBE buffer at 4°C, and the fraction of single- and double-stranded RNA was quantified by densitometry.

### RNA unwinding

RNA unwinding reactions with subsequent separation of 32/9mer and released (3′-fluorescently labeled) 9mer by native PAGE were performed with 10 µM Hera and 5 µM 32/9mer in 50 mM Tris/HCl, pH 7.5, 150 mM NaCl, 5 mM MgCl_2_ in the presence of 5 mM ATP at 25°C, and stopped and analyzed as described (7).

RNA unwinding was followed in real time using a fluorescence resonance energy transfer (FRET)-based assay. The 32/9mer substrate was generated by incubating a 2-fold molar excess of a 3′-Cy3-labeled 9mer with the 5′-Cy5-labeled 32mer in 50 mM HEPES/NaOH, pH 7.5, at 25°C for 1 h. Unwinding reactions were performed in 50 mM Tris/HCl pH 7.5, 150 mM NaCl and 5 mM MgCl_2_ at 25°C with 0.5 µM RNA substrate, 5 µM Hera, Hera_core or Hera_RBD, 2 mM ATP and 5 µM unlabeled 9mer RNA as a trap to prevent re-annealing of labeled RNA after strand separation. For unwinding assays with Hera and Hera_core, protein and ATP were added sequentially, whereas Hera_RBD was added simultaneously with ATP. The donor (Cy3) was excited at 554 nm (1 nm bandwidth), and unwinding was followed as a decrease in acceptor (Cy5) emission at 666 nm (2 nm bandwidth). Unwinding experiments where substrate and product were separated by native PAGE and with direct detection of unwinding by FRET determined similar unwinding rates of 12 × 10^−^^3^ s^−^^1^ and 14 × 10^−^^3^ s^−^^1^, confirming that the decrease in FRET and in acceptor fluorescence is indeed due to strand separation (Supplementary Figure S7).

### CD spectroscopy

Far-UV circular dichroism (CD) spectra of 10 µM Hera, 10 µM Hera_RBD and 10 µM RNA in 50 mM potassium phosphate buffer, pH 7.5, were measured at 25°C from 200 to 260 nm in 1 nm steps with an integration time of 5 s/nm in a 0.1 mm quartz cuvette in an Aviv 62A DS CD spectropolarimeter, accumulated 5-fold and corrected for buffer contributions.

### NMR experiments

NMR spectra were recorded with BRUKER Avance 600, 700 and 950 MHz spectrometers equipped with triple resonance cryogenic probes in 25 mM Bis-Tris/HCl, pH 6.0, 50 mM NaCl, 10% (v/v) D_2_O at 37°C and Hera_RBD concentrations of 55 or 100 µM (titration experiments), or 300 µM (triple resonance experiments). HNCO, HNCACB and HNCA spectra of Hera_RBD and the RBD/21mer complex (RNA:protein 1.33:1) were recorded at 37°C using standard pulse sequences (28). NMR data were processed using the Bruker Topspin 2.1 software and analyzed with CARA (29).

CSP values were calculated from the difference in ^1^H (ΔH) and ^15^N (ΔN) chemical shifts according to eq. 2:
(2)
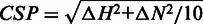



K_d_ values of RNA/RBD complexes were determined from the concentration dependence of the CSP using eq. 1 (with CSP instead of r, and ΔCSP_max_ instead of ΔF_max_, and an offset r_0_ of zero). CSPs of at least 10 amino acids located along the entire protein sequence were analyzed independently.

### Structure determination of Hera_RBD_K463A, Hera_RBD_R444A/R449A and of the Hera_RBD/RNA complex

Crystallization was performed at 20°C in the sitting drop vapor diffusion setup. A stoichiometric Hera_RBD/RNA complex at 0.45 mM concentration was formed by mixing 6.1 µl of 1.47 mM Hera_RBD in 50 mM Tris/HCl, pH 7.5, 500 mM NaCl with 9 µl of 1 mM 19mer RNA, 4.8 µl water and 0.1 µl of 2 M MgCl_2_. Crystals were obtained by 1:1 (v/v) mixing of the complex with reservoir solution containing 1.06 M Na-malonate, pH 6.0, 0.1 M Tris/HCl, pH 7.5, 0.13 M K/Na-phosphate and incubation for 3 months during which time the RNA decomposed. The Hera_RBD_R444A/R449A double mutant was crystallized by diluting a 2.1 mM protein solution in 50 mM Tris/HCl, pH 7.5, 500 mM NaCl with water to a concentration of 462 µM and 1:1 (v/v) mixing with un-buffered reservoir solution containing 26% PEG8000 and 0.1 M Zn(OAc)_2_. Hera_RBD_K463A was crystallized by mixing a 750 µM protein solution in 25 mM Tris/HCl, pH 7.5, 250 mM NaCl 1:1 (v/v) with 3.2 M ammonium sulfate and 0.1 M Bicine/HCl pH 9.0. Crystals (except those of the double mutant) were cryoprotected with paraffin oil and embedded in a vitreous matrix by hyperquenching (30). Data were collected at Swiss Light Source beamline PX-I (RNA complex) and PX-III (mutants), integrated with XDS (31) and scaled with SADABS (Bruker; RNA complex and double mutant) or SCALA (32) (K463A mutant). *Thermus thermophilus* Hera_RBD (PDB-ID 1I31) was used as the search model for molecular replacement with PHASER (33). Models were built in COOT (34) and refined with PHENIX (35). The trigonal data for the K463A mutant are perfectly merohedrally twinned with a twin law (-h,-k,l), emulating a hexagonal lattice. The trigonal asymmetric unit contains three molecules, excluding higher symmetry owing to packing considerations. Refinement for this model was carried out using the protocols for twinning and NCS restraints. For two of the three molecules, the C-terminal sequence could be traced entirely to aa 510. The C-terminal part of the third molecule is disordered beyond aa 503. Data collection and refinement statistics are summarized in Table 1. Figures were created with Pymol (www.pymol.org).

**Table 1.** Data collection and refinement statistics

Data set	R463A, 4I69	R444/449A, 4I68	RNA-complex, 4I67
Construct	425-510	421-510	424-499
Data collection	78.8–1.8	46.3–1.63	44.2–2.3
Resolution range (Å)^a^	(1.9–1.8)	(1.72–1.63)	(2.4–2.3)
Space group	P3_1_	P2_1_2_1_2_1_	P6_1_
Cell dimensions (Å)	a = 49.2, c = 78.8	a = 25.2, b = 46.0, c = 59.6	a = 88.4, c = 28.9
Unique reflections	19 275 (2381)	9118 (1292)	5963 (714)
Multiplicity	3.3 (1.7)	6.4 (4.0)	9.7 (9.9)
Completeness (%)	95.6 (78.5)	99.4 (97.5)	99.9 (99.4)
R_sym_ (%)^b^^,^^c^	9.2 (58.7)	15.3 (63.5)	5.2 (86.8)
Average <I/σ(I)>^b^	8.9 (1.1)	8.5 (1.4)	19.1 (1.2)
Refinement	42.7–1.8	36.4–1.63	44.2–2.3
Resolution range (Å)	(1.9–1.8)	(1.87–1.63)	(2.9–2.3)
R_cryst_ (%)^c^	20.4 (32.1)	18.2 (22.5)	18.0 (27.6)
R_free_ (%)^c^	24.5 (32.5)	23.1 (26.1)	23.4 (36.0)
Number of residues/H_2_O/nucleotides	252/65/–	90/111/–	76/11/4
Rmsd bonds/angles (Å/°)	0.010/1.3	0.006/1.2	0.007/1.1
Ramachandran plot (%)^d^	97.6/2.4	98.7/1.3	94.6/5.4

^a^Values in parentheses correspond to the highest resolution shell.

^b^Calculated with XPREP (Bruker).

^c^R-factor definitions as summarized in (36). R_free_ (37) is R_cryst_ with 5% of test set structure factors.

^d^Calculated using COOT (34). Numbers reflect the percentage amino acid residues of the preferred and allowed regions.

## RESULTS

### RBD is the major determinant for RNA binding to Hera

**Figure 2.**
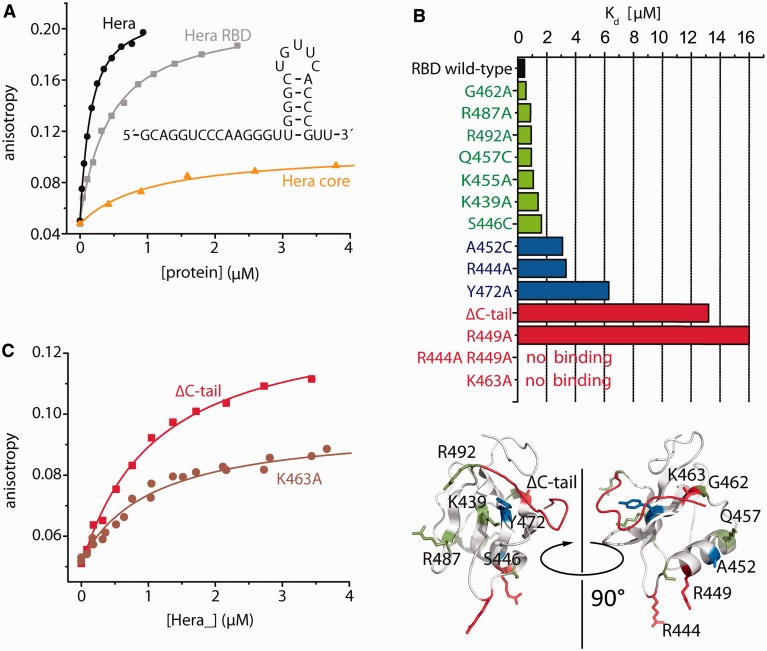
Contributions of Hera domains and individual RBD residues to RNA binding. (**A**) Contributions of domains to RNA binding. Fluorescence anisotropy titrations of a 32mer RNA (inset) with Hera, Hera_core and Hera_RBD. The K_d_ values are 0.13 ± 0.07 µM (Hera), 0.45 ± 0.036 µM (GST-Hera_RBD) and 1.0 ± 0.18 µM (Hera_core), identifying the RBD as the major determinant for RNA binding in the absence of ATP. (**B**) Mutational analysis of the RBD. Positively charged surface residues on the RBD surface (R444, R449, K455, K463, R487 and R492), and two conserved residues on the central β-sheet (K439 and Y472) were changed to alanines, residues on α1 (S446, A452, Q457) and on β1 (G462) were replaced by cysteines, or the C-terminal tail (aa 492-510) that contains six arginines (R492, R493, R503, R505, R506 and R509) was deleted (ΔC-tail). K_d_ values of complexes with the 5′-fluorescein-labeled 32mer RNA were determined for all mutants in fluorescence anisotropy titrations. According to the K_d_ values, the variants can be grouped into three classes: (1) with no or only a moderate (<5-fold) effect on RNA binding (K439A, S446C, G452C, K455A, Q457C, R487A and R492A, green), (2) with an intermediate (5- to 10-fold) effect on RNA binding (R444A, A452C and Y472A, blue) and (3) mutants that showed a >25-fold increase in the K_d_ values (R449A, R444/449A, K463A and ΔC-tail, red). (**C**) Fluorescence anisotropy titrations of the 5′-fluorescein-labeled 32mer RNA with Hera_K463A and Hera_1-491 lacking the C-terminal tail. With 1.0 ± 0.17 µM (_K463A) and 1.1 ± 0.13 µM (_1-491), the K_d_ values are identical to the value for the Hera_core/32mer complex, indicating that the RBDs do not contribute to RNA binding in these mutants. Titrations were performed in 50 mM Tris pH 7.5, 150 mM NaCl and 5 mM MgCl_2_ at 25°C with 50 nM 5′-fluorescein-labeled 32mer RNA. Fluorescence was excited at 495 nm and detected at 530 nm.

Hera consists of a helicase core formed by two RecA domains (RecA_N, RecA_C), a DD and a C-terminal RBD ((14–18); Figure 1). We have previously shown that Hera binds and unwinds a minimal RNA substrate formed by a 32mer containing hairpin 92 of 23 S ribosomal RNA, annealed to a 9mer complementary to the flanking single-stranded RNA region upstream (5′) of the hairpin (7). To dissect contributions of individual domains of Hera to RNA binding, we first determined dissociation constants of the complexes of Hera, Hera_core and the isolated Hera-RBD with the 5′-fluorescein-labeled 32mer in fluorescence anisotropy titrations (Figure 2A) in the absence of ATP. To increase the anisotropy change on binding, a GST-fusion protein of the RBD was used in these experiments. The dissociation constants determined were K_d_ = 0.13 ± 0.07 µM (Hera), K_d_ = 0.45 ± 0.036 µM (GST-Hera_RBD) and K_d_ = 1.0 ± 0.18 µM (Hera_core). These values are in agreement with RBD as the primary RNA-binding site of Hera, with minor contributions from the helicase core in the absence of ATP. To test for possible contributions of the DD to RNA binding, we also performed titrations with Hera_370-510 (DD, RBD) and with Hera_208-419 (RecA_C, DD; Supplementary Figure S2A). Whereas the Hera_370-510/RNA complex showed a similar K_d_ as observed for the RBD only, the K_d_ for the Hera_208-419/RNA complex was increased ∼70-fold. Thus, the DD or the RecA_C domains do not contribute to RNA binding to Hera. Overall, our results demonstrate that Hera_core has a low affinity for (single-stranded) RNA, and the RBD confers high-affinity RNA binding to Hera in the absence of ATP. Stoichiometric titrations (Supplementary Figure S2B) are consistent with a 1:1 stoichiometry of 32mer RNA per Hera protomer, or per Hera_RBD.

### Mutational analysis of RNA binding to the RBD

**Figure 3.**
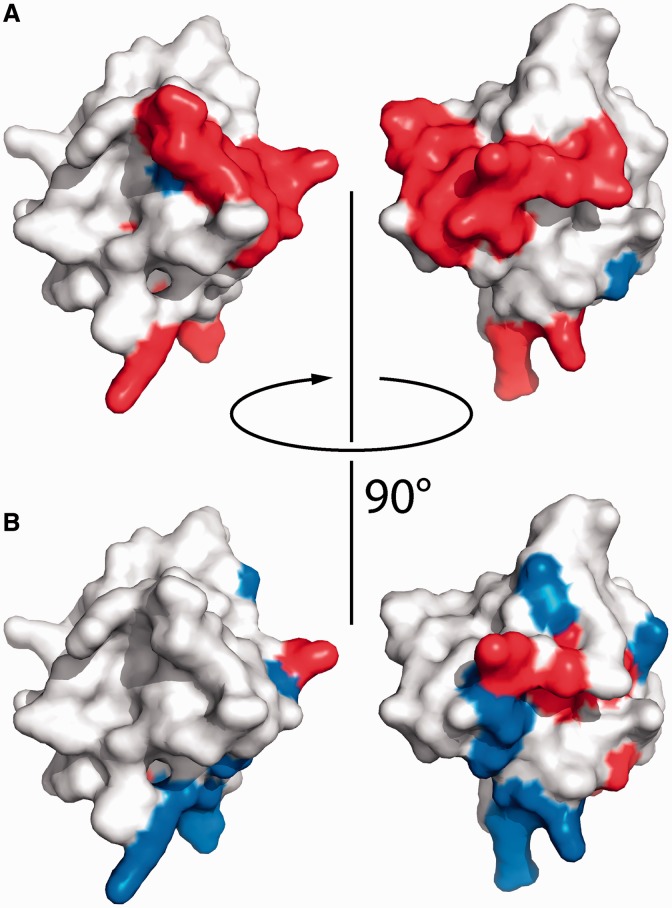
Residues involved in RNA-binding cluster in two regions. Mapping (**A**) the effect of mutations on RNA affinity or (**B**) the CSP of individual amide protons on RNA binding on the RBD structure (Hera_RBD_K444/449A, PDB-ID 4I68) reveals two clusters, one involving α-helix α1 and β-strand β2 and a second involving the C-terminal tail. The color code for the effect of mutation on RNA affinity is the same as in Figure 2B. Residues with CSP values of 0.3–0.5 are colored in blue, residues with CSP values > 0.5 in red.

The RBD of Hera adopts a modified RRM fold (15). RRMs show different modes of RNA binding (reviewed in (23,38)). RNA can bind diagonally across the central β-sheet by interactions with conserved residues on one side of the β-sheet; or perpendicular to the strands of the β-sheet, involving residues form the β-sheet and a loop; or by only contacting loop regions. The RNA-binding mode of Hera_RBD could not be inferred from the crystal structure (15). To identify the residues involved in RNA binding to the Hera RBD, we mutated positively charged surface residues (R444, R449, K463, R487 and R492), and the two conserved residues on the central β-sheet (K439 and Y472) that have been demonstrated to contribute predominantly to the classical mode of RNA binding (38). We also deleted the C-terminal tail (aa 492-510; ΔC-tail) that contains six arginines (R492, R493, R503, R505, R506 and R509) and was disordered in the Hera_RBD crystal structure (15). K_d_ values of complexes with the 32mer RNA were determined for all mutants in fluorescence anisotropy titrations (Figure 2B). Mutants were grouped into three classes according to their K_d_ values: (i) with no or only a moderate (<5-fold) effect on RNA binding (K439A, R487A and R492A), (ii) with an intermediate (5- to 10-fold) effect on RNA binding (R444A and Y472A) and (iii) mutants that showed a >25-fold increase in the K_d_ values (R449A, R444/449 A, K463A and ΔC-tail). On the RBD surface, the residues involved in RNA-binding are clustered in two regions, one involving α-helix α1 and β-strand β2, and one formed by the C-terminus (Figure 3A).

### Mapping the RNA-binding site by NMR CSP experiments

**Figure 4.**
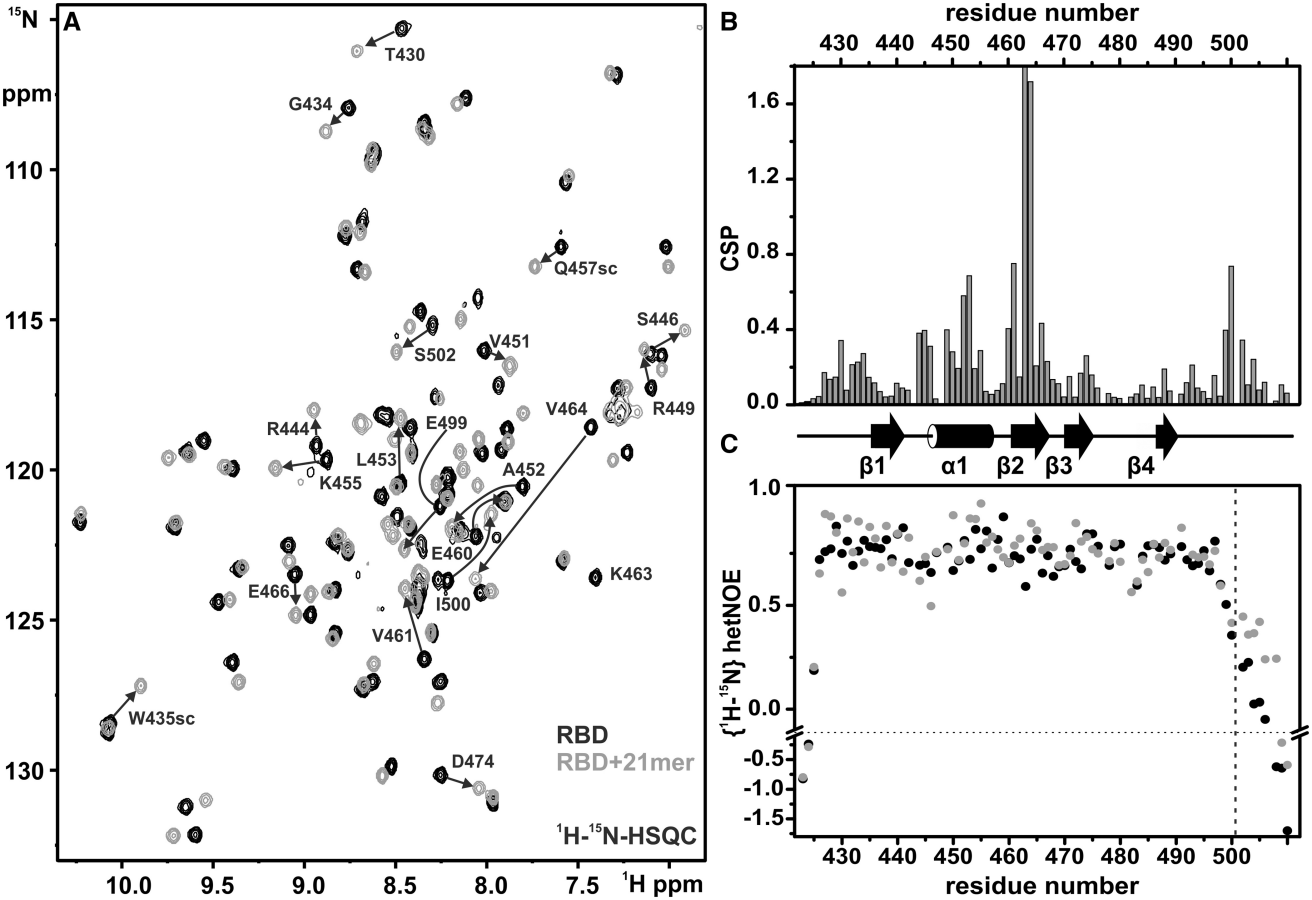
NMR characterization of the Hera-RBD/RNA interaction. (**A**) Overlay of ^1^H,^15^N-HSQC spectra of the Hera-RBD in its free form (black) and in the presence of saturating amounts (∼1.5 equiv.) of the 21 mer RNA (gray). Assignments are indicated for those amide groups that show the largest CSPs on RNA binding. The direction of the shift changes are given by black arrows. (**B**) Combined ^1^H and ^15^N chemical shift changes of the Hera-RBD on 21mer RNA binding plotted against its amino acid sequence. The cartoon below the plot shows the location of secondary structure elements along the sequence. (**C**) Changes in the {^1^H}-^15^N-HetNOE for the Hera-RBD on 21mer RNA binding (black: free Hera-RBD, gray: protein/RNA complex). The start of the C-terminal area for which the most pronounced changes are observed is marked by a dashed line. NMR measurements were performed in 25 mM Bis-Tris/HCl, pH 6.0, 50 mM NaCl and 10% (v/v) D_2_O at 37°C.

NMR CSP experiments allow mapping of regions involved in RNA binding through changes in amide proton chemical shifts on RNA binding. A ^1^H,^15^N-HSQC spectrum of the ^15^N-labeled Hera RBD (Figure 4A) shows well-defined resonances with a high dispersion, as expected for a structured protein. Chemical shifts of all backbone amide protons, of nitrogen atoms in non-proline amino acids and of 96% of the backbone carbon atoms could be assigned from the standard set of triple resonance experiments (Supplementary Figure S3A). The unassigned resonances correspond to the seven carbonyl carbon atoms of amino acids preceding a proline, and one C_β_-signal (P481). From the assigned H^N^-, N^H^-, C_α_-, C_β_- and CO chemical shifts, the chemical shift index was calculated with PECAN (22) (Figure 4B). Overall, the positions of secondary structure elements assigned from the chemical shift indices largely agree with the secondary structure elements present in the crystal structure (15) (Supplementary Figure S4A). The N-terminal sub-domain exhibits a moderate propensity for β-sheet formation, helix α2 present in canonical RRMs is absent and the chemical shift index values of the C-terminal tail following β4 are in agreement with random coil conformation. Thus, the structural features that set the Hera RBD apart from canonical RRM folds are also evident in solution.

{^1^H}-^15^N-Hetero-NOE (HetNOE) experiments provide information on protein dynamics, with HetNOE-values of >0.5, indicating conformational rigidity. For Hera_RBD, high HetNOE values are indicative of a rigid peptide backbone from aa 425 to 499 (Figure 4C), a region that comprises the N-terminal sub-domain preceding β1, the long loop connecting β3 and β4 and the first part of the C-terminal tail following β4 (aa 490-499). In agreement with the HetNOE data, the C-terminus beyond aa 499 was disordered in the first Hera_RBD crystal structure (15), but can adopt different conformations (see later in the text).

To identify possible RNA-binding sites on the Hera_RBD, CSP experiments were conducted. Binding of the 32mer led to severe line broadening and disappearance of the majority of the resonances from the RBD, precluding the identification of CSP effects for individual amide protons (data not shown). In contrast, the RBD complex with a shortened 21mer that comprises the stem-loop region but lacks the largest part of the 5′-single stranded region yielded high-quality spectra (Figure 4A and Supplementary Figure S3), despite an intermediate to fast exchange on the NMR timescale. In titration experiments, large changes in chemical shifts were observed for a number of resonances, saturating at a ∼1.5:1 ratio of RNA to protein. From the concentration dependence of chemical shifts, a K_d_ of ∼6 µM (at 37°C, pH 6 and 50 mM NaCl) can be estimated. K_d_ values from electrophoretic mobility shift assay and isothermal titration calorimetry experiments (at 25°C, pH 7.5 and 150 mM NaCl, Supplementary Figure S2C and S2D) are somewhat higher (20–40 µM), possibly because of the higher ionic strength. Interestingly, the resonance for K463 disappears in the RBD/RNA complex, possibly owing to exchange effects. Mutation of K463 abolishes RNA binding (see previously). From HSQC spectra of the RBD and the RBD/21mer complex, in combination with an HNCA experiment for the RBD/21mer complex, all backbone amide signals (except K463) for the RBD bound to RNA were assigned. Large changes in chemical shift were identified for residues in the N-terminal sub-domain (aa 425-435), in α-helix α1 (aa 443-454), in β-strand β2 (aa 460-467) and in the C-terminal tail (aa 498-504) (Figure 4B). The directions of the CSPs are shown in Supplementary Figure S3B. When mapped on the surface of the RBD (Figure 3B), regions exhibiting changes in chemical shifts in the RBD/RNA complex are in agreement with the two clusters identified by the mutational analysis (Figure 3A).

The changes in chemical shifts, as well as the mutational analysis, indicate a crucial role of residues in helix α1 for RNA binding. We therefore probed the contributions of S446, A452 and Q457, all located within this helix, to RNA binding in anisotropy titrations of the RBD carrying the S446C, A452C, K455A and Q457C mutations (Figure 2B). Only the mutation of A452 had an effect on RNA binding (10-fold increase in K_d_), suggesting that α-helix α1 mainly contacts the RNA through the positively charged side-chains of R444 and R449.

### Contributions of RBD residues to RNA binding in the context of full-length Hera

We also probed the effect of the K463A mutation and of the deletion of the C-terminal tail in the context of full-length Hera. For the Hera_K463A/32mer and Hera_1-491/32mer complexes, the K_d_ values were 1.0 ± 0.17 µM and 1.1 ± 0.13 µM (Figure 2C). These values are identical to the K_d_ value of the Hera_core/32mer complex, demonstrating that the RBD does not contribute to RNA binding in these mutants. These results underline the importance of K463 and the C-terminal tail for RNA binding to the RBD.

### Role of the C-terminal tail

**Figure 5.**
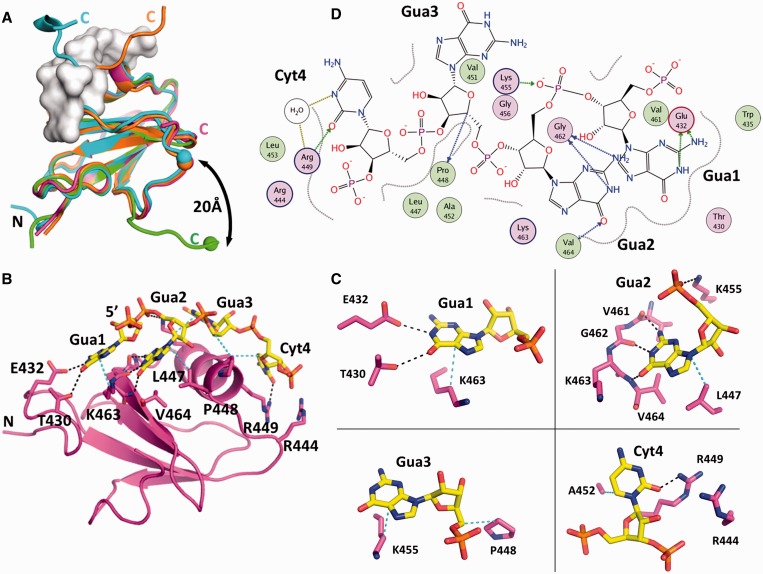
Crystal structure of the Hera_RBD/RNA complex. (**A**) Superposition of wild-type Hera_RBD structure (green, PDB-ID 3i31) with the mutant K463A (orange), the double mutant R444A/R449A (cyan) and the RNA complex (magenta). The RNA is represented as a gray surface. The C-terminal part of the RBD, starting from residue R492, can adopt several conformations. In the wild-type structure, the C-terminus (**C**) is swung away from the RBD, whereas in the mutants, it interacts with the RBD, in agreement with the NMR data. The end of the C-terminus blocks the RNA-binding site and has to move away for RNA binding (clashes with the gray surface). The magnitude of the required movement is indicated by spheres for Gly496 in each structure. This part of the Hera_RBD C-terminus is not visible in the Hera_RBD/RNA complex. Note that the C-termini of the K463A and R444A/R449A mutants adopt the same conformation up to Arg503, after which they diverge. The mutations do not influence the conformation of the C-termini. (**B**) Overview of the RNA-binding site. Possible hydrogen bonds and van der Waals interactions are drawn as dashed black and cyan lines, respectively. The small N-terminal sub-domain (N) of the RBD contributes to binding of Gua1. (**C**) Details of the RNA-binding site, showing individual bases and their interaction with RBD residues. The color code is the same as in (B). (**D**) Schematic drawing of the RNA/RBD interactions. A two-dimensional projection of the RNA is shown with polar atoms color-coded. Protein residues with polar and hydrophobic interactions are shown as magenta and green circles, respectively. Possible hydrogen bonds are depicted as blue arrows. The wavy lines around the RNA delimit areas that are contacting the RBD and are thus shielded from solvent.

The deletion of the C-terminal tail (aa 492-510) led to a 25-fold decrease in 32mer affinity, suggesting an important role of this region in RNA binding to the Hera RBD. In the crystal structure of the RBD (15), residues 493-498 were distended from the RBD core (see Figure 5A), and HetNOE NMR experiments are in agreement with a flexible C-terminal tail (aa 500-510, Figure 4C).

To visualize the structure of the C-terminal tail of the Hera_RBD, we crystallized several constructs and mutants of Hera_RBD. Two of the mutants, Hera_RBD_K463A and R444A/R449A, yielded structures with their entire C-terminal sequences visible in the electron density maps up to aa 510. In all structures where the C-terminal part is ordered, residues 493-498 fold back onto the outermost β-strand β_2_ of the RBD and physically block the RNA-binding site (cyan and orange in Figure 5A), with exception of the wild-type Hera_RBD structure. Although the point mutations are likely responsible for the different crystal forms, they do not *per se* impose this particular conformation of the C-terminus, and only the far ends of the C-termini extending away from the RBD are dictated by crystal contacts. The C-terminal part of the RBD can therefore adopt at least two major orientations, distended from the RBD or attached to the β_2_ strand. The last residue visible in the distended conformation is Leu498, the C_α_ atom of which is 22 Å away from its position in the ‘attached’ conformation. In solution, this region might adopt a multitude of conformations, which is supported by the presence of low HetNOE values beyond residue 499 (see previously). Sizeable movements of the C-terminal region would also be in agreement with the observed large changes in chemical shifts for residues 498-504 on RNA binding (see previously). In the crystal structures, the proline peptide bonds at P501 and P507 are in the trans conformation, although ^1^H,^15^N-HSQC-spectra showed two signals with different intensities for some amino acids in the C-terminal tail, suggesting that both cis- and trans-conformations can be populated (Supplementary Figure S3). Altogether it can be concluded that the C-terminus of Hera_RBD exhibits considerable conformational plasticity.

To investigate whether the C-terminal tail adopts a defined structure on RNA binding, we measured far-UV CD spectra of Hera_RBD, the 32mer and a RBD/32mer complex (Supplementary Figure S4B). However, the spectrum of the complex was virtually identical to the sum of the individual spectra (Supplementary Figure S4C). Similarly, the chemical shift indices of amino acids in the C-terminal tail are similar for Hera_RBD and the RBD/21mer complex (Supplementary Figure S4A). Altogether, these findings imply a possible direct role of the C-terminal tail in RNA binding, albeit without the formation of a regular secondary structure in the RBD/RNA complex. The HetNOE values of the C-terminal amino acids in the RNA/complex do not reach the values typical of rigid conformations, indicating that the interaction between the C-terminus of the RBD and the RNA is dynamic in nature and most likely based on unspecific electrostatic interactions.

### Crystal structure of a Hera_RBD/RNA complex

Hera_RBD/RNA complexes prepared for structure determination included wild-type Hera_RBD that extended all the way to the C-terminal residue 510 or was C-terminally truncated at residue 491, and the 32mer or 19mer RNAs as binding partners. Crystals of Hera_RBD (residues 424-510) in complex with a tetranucleotide were obtained and the structure of the complex was determined to 2.3 Å resolution. The tetranucleotide must have been generated *in situ* by partial hydrolysis of the 19mer RNA. Based on clear electron density and hydrogen bonding distances, the sequence of the RNA could be assigned as 5′-GGGPyr-3′. The only GGGPyr element present in the 19mer RNA is a GGGC stretch, and GGGC was therefore modeled into the electron density.

The RBD core adopts a similar structure in the RNA-free and RNA-bound form (Figure 5A), in agreement with the secondary structure information from NMR (Supplementary Figure S4A). In the RBD/RNA complex structure, the C-terminal tail is visible up to aa 499, whereas the remainder appears to be disordered. Residues 492-499 pack closely to the RBD without engaging in contacts with the bound RNA (Figure 5A and B). This ‘closed’ conformation is consistent with higher HetNOE values observed for these residues in NMR experiments (see previously) that indicate rigidity. For RNA to bind to Hera_RBD, the C-terminal part (aa 502-510) has to move away by at least 8 Å compared with the structure where this part is attached to the RBD (Figure 5A).

Individual interactions involved in RNA binding to Hera_RBD are summarized in Supplementary Table S1. The 5′-guanine (Gua1) of the GGGPyr sequence is base-specifically bound by the N-terminal subdomain of Hera_RBD, a small addendum of 12 residues (A424-W435) unique to Hera (Figure 5B–D). The side-chains of T430 and E432 within this sub-domain form hydrogen bonds with the Watson–Crick face of Gua1. Further, the side-chain of K463 stacks against the side of Gua1, rationalizing the deleterious effect of the K463A mutation on RNA binding to the RBD (see previously). Thus, Gua1 seems to be specifically tied to the N-terminal sub-domain of Hera_RBD. The second nucleotide, Gua2, binds through three hydrogen bonds of the Watson–Crick face to main-chain amide groups (V461, G462 and V464, Figure 5B–D). The main-chain interaction of G462 with Gua2 is consistent with appreciable CSPs of G462 on RNA binding, but a negligible effect of its mutation on RNA affinity. The interaction pattern nicely explains the specificity for guanine at this position. The third nucleotide, Gua3, forms no base-specific interactions with Hera_RBD (Figure 5B–D). The driving force for the binding of Gua3 seems to be a hydrophobic interaction with the side-chain of K455. The ammonium group of K455 engages in a salt bridge with the 5′-phosphate group of Gua2. In agreement with the role of K455 in RNA binding, it experiences a significant change in chemical shifts, although the effect of mutating K455 to alanine is moderate. Finally, the fourth nucleotide, Pyr4, packs against A452 and L453 (Figure 5B–D), explaining the increase in K_d_ on A452 mutation. The 3′-phosphate of Pyr4 interacts with the R444 side-chain.

To test whether the 4mer RNA observed in the crystal structure recapitulates binding to the Hera_RBD, we determined the K_d_ value of the 4mer/RBD complex in anisotropy titrations (Supplementary Figure S5). The K_d_ value was 11 µM. For the Hera_RBD_ΔC-tail/32mer RNA complex, a similar K_d_ of 13 µM was determined (Figure 2B), demonstrating that the 4mer does not contact the C-tail, but otherwise forms the same interactions with the RBD as the 32mer. The GGGC stretch in the 19mer RNA is part of the hairpin stem that, according to NMR results (see later in the text), is indeed formed in solution. The GGGC sequence bound to the RBD thus must have become available for binding upon degradation of the 19mer RNA. In the 21mer and 32mer RNA, a GGGU sequence is present outside the stem in the single-stranded region directly 5′ of the hairpin. These RNAs most likely bind to the RBD core through the GGGU sequence, with the hairpin remaining intact.

**Figure 6.**
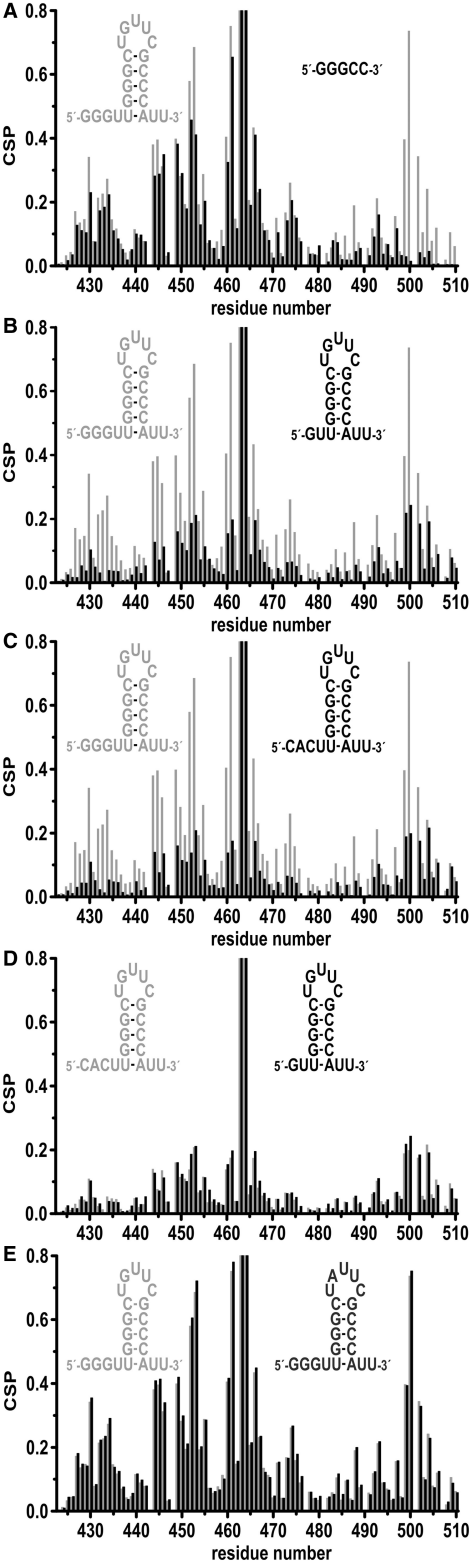
Differences in RNA binding as assessed by CSP experiments. Comparison of the combined chemical shift changes (^1^H,^15^N) caused by (**A**) the 21mer RNA (gray) and the 5mer 5′-GGGCC-3′ (black), (**B**) the 21mer RNA (gray) and a 19mer RNA (black) lacking the two 5′-guanine nucleotides, (**C**) the 21mer RNA (gray) and a modified 21mer RNA (black) where the three 5′-guanine nucleotides are replaced by a CAC-sequence, (**D**) the 19mer RNA (black) and the modified 21mer RNA (gray) and (**E**) the 21mer RNA (gray) and the 21mer_modloop where the G in the apical loop has been replaced by an A (black).

To further confirm that the binding mode of the short oligonucleotide in the crystal structure reflects binding of larger RNAs to Hera_RBD, CSP experiments were performed with a 5mer RNA (5′-GGGCC-3), similar to the RNA in the crystal, that includes the 3′-phosphate of Cyt4. For residues in the RBD core (aa 424-499), changes in chemical shifts of amide protons on RBD/5mer complex formation are similar to the ones observed for the RBD/21mer complex, both in magnitude and pattern (Figure 6A and Supplementary Figure S3B), suggesting a similar binding mode of both RNAs to this part of Hera_RBD. However, in contrast to the 21mer, binding of the 5mer RNA to the RBD does not induce changes in the chemical shifts of amide protons from residues in the C-terminal tail (aa 500-510, Figure 6A), in-line with this short RNA not extending from the central binding site to reach the C-terminus. In agreement with this finding, the affinity of Hera_RBD for this short single-stranded RNA is independent of the presence of the C-tail (Supplementary Figure S5). Overall, the crystal structure thus captures an RBD/RNA complex that reflects Hera’s natural preference for RNA substrates. The N-terminal sub-domain creates a specific guanine binding pocket, and sets Hera apart from RBDs of other helicases (see Discussion).

### RNA elements contacting the RBD

**Figure 7.**
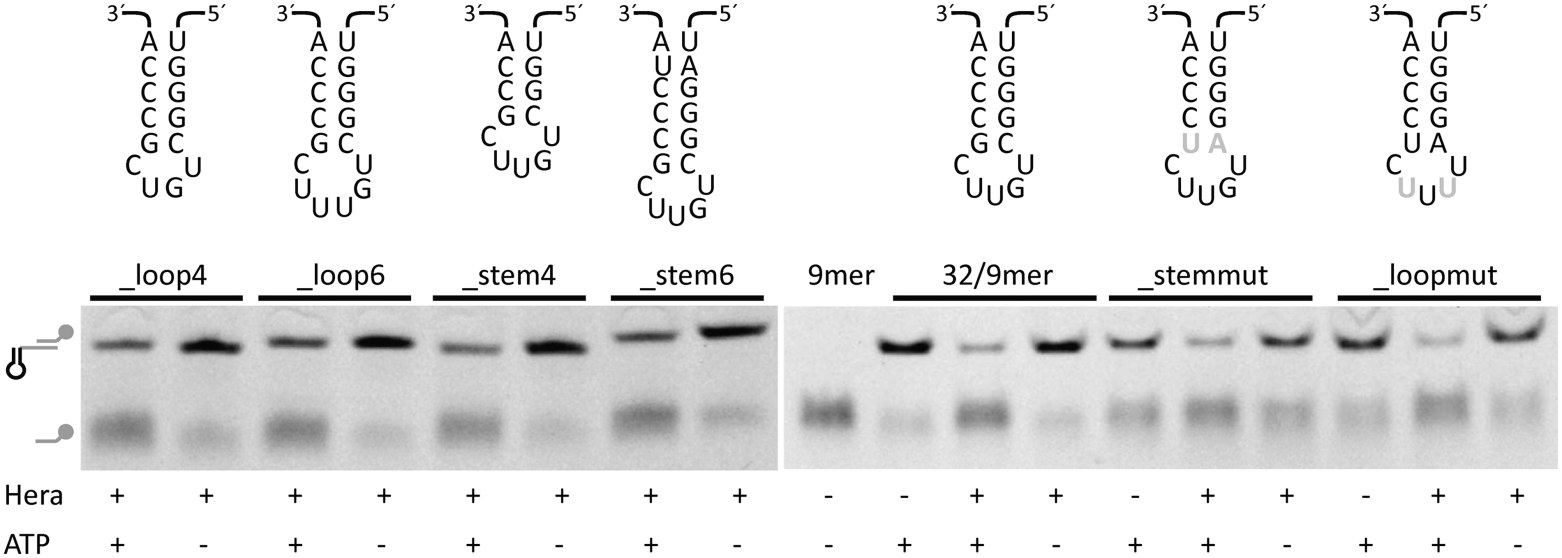
RNA unwinding of modified 32/9mer RNA substrates by Hera. Top: Modifications introduced into the 32mer included shortening or extending the stem by 1 bp (_stem4, _stem6), altering a base-pair in the stem (_stemmut), shortening or expanding the loop by one nucleotide (_loop4, _loop6), replacing the G and one of the Us in the loop (_mutloop). Mutated bases are highlighted in gray. For clarity, the adjacent duplex formed by annealing of the 9mer has been omitted Bottom: Unwinding of the 32/9mer RNA and its variants by Hera. The reactions were performed in 50 mM Tris pH 7.5, 150 mM NaCl and 5 mM MgCl_2_ at 25°C in the presence of a 10-fold excess of non-labeled trap strand to ensure single turnover conditions. Reactions were stopped after 30 min, and double-stranded substrate and released single-stranded 9mer were separated by PAGE (see cartoon, the duplex that is unwound is shown in gray). All RNAs were unwound in the presence of ATP, implying that neither the length nor sequence of the loop nor the exact length of the stem is critical for binding of these RNA to the RBD.

In addition to changes in chemical shifts of RBD residues, NMR experiments can also provide structural information on RNA elements involved in binding to Hera_RBD through resonances for imino protons, typically found between 10 and 15 ppm. The 1D ^1^H-spectrum of the 21mer RNA contains four peaks in the region between 11.6 and 13.5 ppm, indicative of the presence of four G-C base pairs, consistent with the hairpin 92 being formed (Supplementary Figure S6). In the RBD/21mer complex, the resonance at 11.6 ppm shows a slight change in chemical shift, and additional signals are present between 10.0 and 11.5 ppm (Supplementary Figure S6B). In the 1D-HSQC-spectrum of ^15^N-labeled RBD in complex with unlabeled 21mer, and in the 1D- and 2D HSQC-spectra of the reverse complex of ^15^N-labeled 21mer and unlabeled RBD, two of these resonances (at 10.2 and 11.1 ppm) show no spectral correlation to nitrogen atoms in either the RBD or the RNA, implying that they correspond to hydroxyl groups. NOEs between these resonances and resonances of aromatic protons within the RBD suggest that they belong to tyrosine, and experiments with the free RBD confirm that these signals are already present in the absence of the RNA (Supplementary Figure S6C). Notably, one of the additional signals in the complex (at 10.5 ppm) can be assigned to a guanine imino proton that becomes protected from proton exchange with the solvent only on complex formation, pointing towards a direct interaction of this guanine with the RBD (Supplementary Figure S6B). The 21mer contains four unpaired guanines, three at the 5′-end and one in the apical loop. To identify which of these guanines is contacted by Hera_RBD, we performed NMR CSP experiments with a 19mer RNA, lacking two of the terminal guanines, a modified 21mer (21mer_mod) where the three guanines at the 5′-end have been substituted by CAC and a modified 21mer (21mer_modloop) where the guanine in the apical loop has been replaced by an adenine (Figure 6). In general, the observed changes in chemical shift in the RBD/19mer complex are much smaller than in the RBD/21mer complex (Figure 6B), although similar changes in chemical shifts are observed for amide protons in the C-terminal tail. The 21mer_mod causes a similar perturbation of chemical shifts as the 19mer (Figure 6C and D), in-line with a similar binding mode. In contrast, 21mer_modloop, lacking the guanine in the apical loop, elicited a similar pattern in CSPs as the 21mer (Figure 6E), confirming that the G in the 5′-terminal region but not the G in the apical loop is bound by the RBD core. Altogether these data are consistent with a similar binding mode of the 5mer, the 21mer and the 21mer_modloop RNAs (Figure 6A and E), but a different binding mode for the 19mer and the 21mer_mod (Figure 6B–D). From the different elements shared by these RNAs, it can be concluded that the 5mer and the 21mer are bound to the RBD core through their GGGU sequence, located 5′ from the hairpin in the 21mer. In addition, the hairpin in the 21mer contacts the C-terminal tail. In contrast, the 19mer and 21mer_mod lack the single-stranded GGGU sequence. These RNAs contact Hera_RBD through the hairpin and interact mainly with the C-terminal tail of the RBD. Overall, this scenario is in agreement with mutational studies of the hairpin and loop regions with Hera (Figure 7). 32/9mer RNA can be unwound by Hera (7). Modified 32/9mer substrates where one base pair of the stem has been added to or deleted from the stem, or where the G-C base-pair adjacent to the loop has been exchanged to an A-U base-pair, are unwound by Hera (Figure 7), and thus bound by the RBD. Similarly, a modified 32/9mer where a U has been added or deleted in the loop, or where the G and a U in the loop have been exchanged by a U and A, respectively, is unwound by Hera (Figure 7). Thus, neither the loop length or sequence nor the hairpin length or sequence appear to be critical for binding of the 32/9mer to Hera. In addition, these results confirm that the G in the loop is not a prerequisite for RNA binding to Hera.

From the dependence of amide proton chemical shifts on the RNA concentration, a K_d_ of 75 µM can be estimated for the RBD/19mer complex. The 19mer thus binds to the RBD with lower affinity than the 21mer (K_d_ ∼ 6 µM, see previously), in agreement with the absence of one recognition element, the GGGU sequence. Interestingly, two K_d_ values are observed for the 21mer_mod/Hera_RBD complex: for resonances of the RBD core residues (aa 424-493), a K_d,1_ value of 40–70 µM can be inferred, whereas resonances from residues from the C-terminal tail yield a higher K_d,2_ value of 150–300 µM. These two different K_d_ values indicate the 21mer_mod RNA can either interact with the C-terminal tail or contact the RBD core region. Possibly, the G in the loop can bind to the pocket formed by the N-terminal sub-domain in the absence of the preferred GGGPyr sequence.

**Figure 8.**
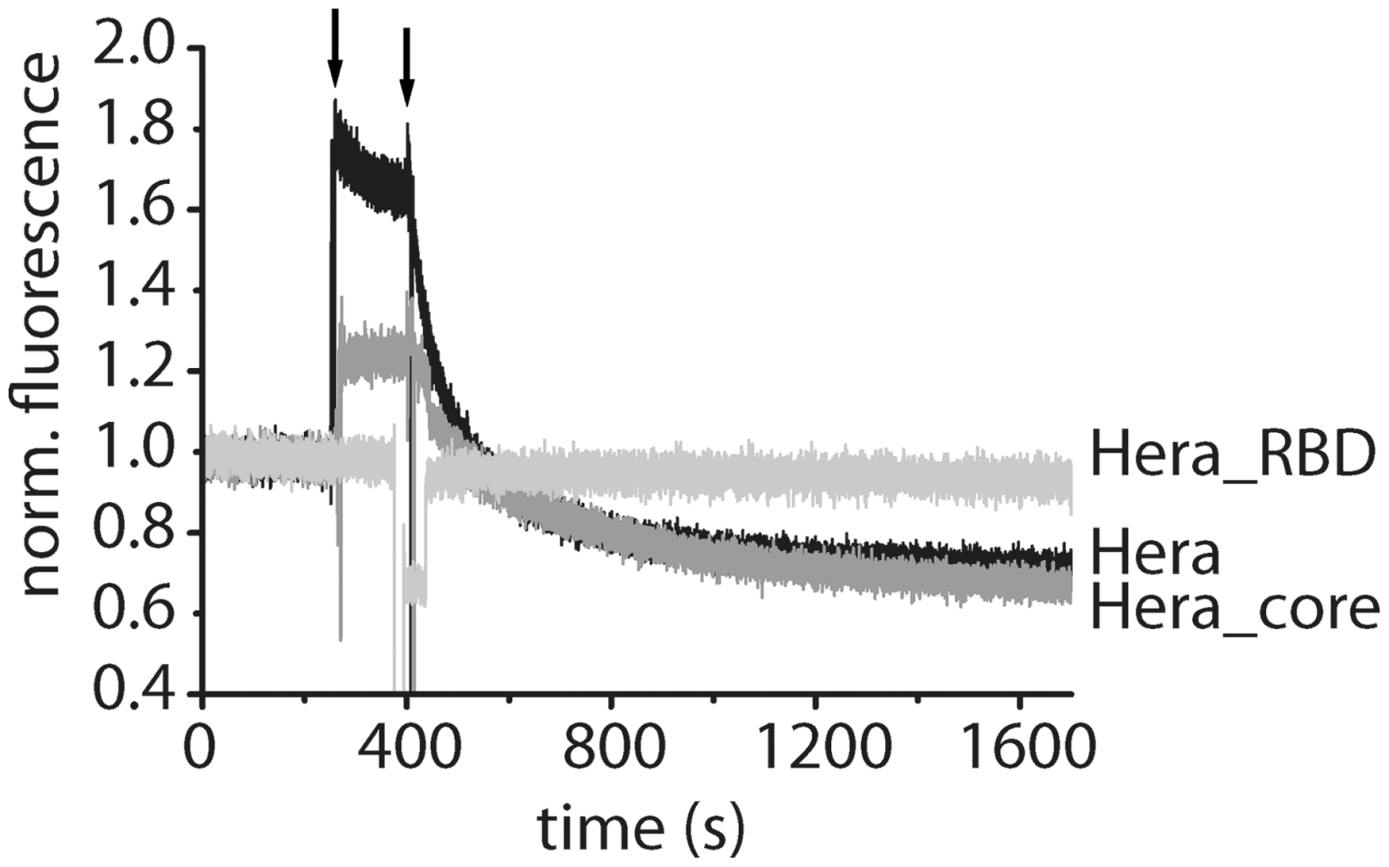
RNA unwinding of the 32/9mer RNA substrate by Hera, Hera_core and Hera_RBD. Unwinding of a FRET-labeled 32/9mer substrate, formed by annealing a 3′-Cy3-labeled 9mer to the 5′-Cy5-labeled 32mer by Hera (black), Hera_core (dark gray) and Hera_RBD (light gray). The initial Cy5 signal of the double-stranded substrate was normalized to 1 for all measurements. The arrows indicate addition of protein (after 250 s) and of ATP (at 400 s) to start the RNA unwinding. Measurements were performed in 50 mM Tris pH 7.5, 150 mM NaCl and 5 mM MgCl_2_ at 25°C in the presence of a 10-fold excess of non-labeled trap strand to ensure single turnover conditions. Cy3 fluorescence was excited at 554 nm, and Cy5 fluorescence was detected at 666 nm. Duplex separation causes a decrease in acceptor (Cy5)-fluorescence for Hera and for Hera_core (see Supplementary Figure 7). The RBD does not show unwinding, indicating that binding of the RBD to single-stranded RNA regions is not sufficient for RNA unwinding.

Overall, our data are thus in-line with the conclusion that the single-stranded GGGU sequence of the 32mer and the 21mer binds to the core of the Hera RBD. This GGGU sequence is immediately adjacent to the double-stranded region of the 32/9mer unwinding substrate. We therefore tested whether binding of the 32/9mer to the RBD already destabilizes the duplex in FRET unwinding assays with Hera, Hera_core and Hera_RBD (Figure 8), where a decrease in acceptor fluorescence because of a decrease in FRET reflects release of the 9mer as a result of unwinding (Supplementary Figure S7). As shown previously (7), Hera readily unwound the RNA substrate. RNA unwinding was also detected with Hera_core, albeit at a reduced rate. In contrast, the RBD alone displayed no RNA unwinding activity. Consequently, the RBD mediates RNA binding to Hera, but duplex separation is performed by the helicase core.

## DISCUSSION

We have shown here that the Hera RBD binds RNA independent of the helicase core, and is the major RNA-binding platform for Hera. We have mapped the regions important for RNA binding to the RBD in a mutational and NMR study to the N-terminal sub-domain, the α-helix α1 and to the C-terminal tail of the RBD. The RBD core region binds single-stranded GGGPyr stretches, as revealed by the crystal structure of the RBD/RNA complex. NMR CSP experiments with different RNA substrates are consistent with the RBD core binding to a single-stranded GGGU region in the RNA substrate, and the C-terminal tail interacting with the stem of an adjacent hairpin. A neighboring duplex can then be unwound by the Hera helicase core in the presence of ATP.

### Comparison of RNA binding by the Hera and YxiN RBDs

**Figure 9.**
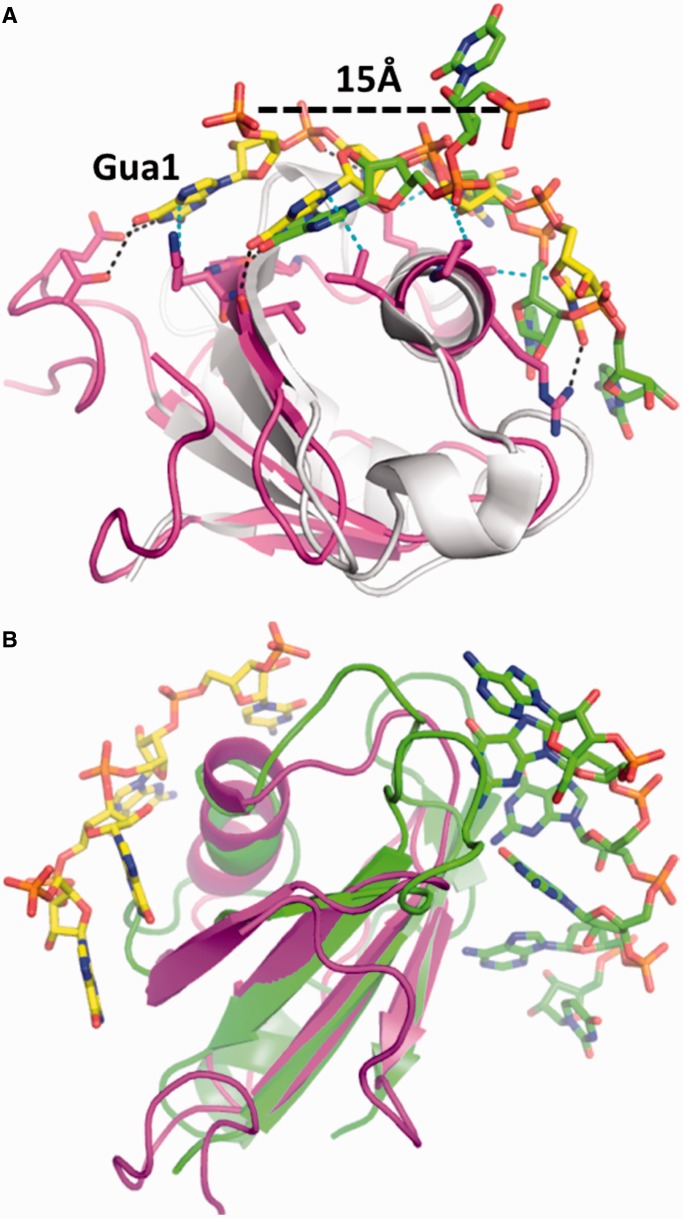
Comparison of the Hera_RBD/RNA complex structure with other RBDs. (**A**) Superposition of Hera_RBD (magenta and yellow) and YxiN_RBD (gray and green) RNA complexes. For the YxiN/RNA complex (PDB-ID 3moj), only an RNA fragment corresponding to that in the Hera_RBD complex is shown. The two central nucleotides correspond well in the structures. The N-terminal sub-domain that is unique to Hera_RBD and binds the 5-guanine base (Gua1) provokes a strong deviation of the RNA path compared with YxiN. The 5′-ends of the corresponding nucleotides are 15 Å apart (dashed line). (**B**) Superposition of the Hera_RBD/RNA complex (yellow) with a quasi-RRM/RNA complex (PDB-ID 2kg1, green). The direction of the two RNAs is consistent with a longer RNA covering both faces of the RBD.

With the 32mer derived from 23 S rRNA, Hera binds to the same RNA as the *B. subtilis* DEAD box protein YxiN (11). However, YxiN recognizes the sequence in the apical loop of hairpin 92 (11,24), whereas Hera recognizes a single-stranded region adjacent to hairpin 92, and only seems to interact with the hairpin through the double-stranded stem. The overall mode of single-stranded RNA recognition by Hera_RBD is similar to YxiN, and both RBDs bind RNA involving the opposite face of the central β-sheet compared with classical RRMs or qRRMs (39) (Figure 9B). The directionality of the single-stranded RNA bound to Hera and to the classical and qRRM would be consistent with the possibility of a longer single-stranded RNA to wrap around the RRM and contact the loops and both faces of the central β-sheet. Although it remains to be seen if such an RNA-binding mode is indeed realized, our results underscore that the versatility of RRMs as RNA-binding platforms is larger than previously thought.

A closer comparison of the two RNA complex structures, however, reveals different sequence specificities (Figure 9A). The only conserved feature between Hera and YxiN is the sequence-specific interaction with the second nucleotide Gua2 that forms three hydrogen bonds through its Watson–Crick face to main-chain amide groups (V461, G462 and V464 in Hera; I436, G437 and I439 in YxiN) in both complexes. In Hera, the N-terminal sub-domain of Hera_RBD (A424-W435) that is not present in YxiN creates a guanine-specific binding pocket for Gua1, with hydrogen bonds between T430 and E432 and the Watson–Crick face of Gua1, and a stacking of the K463 side-chain against the side of Gua1. A pyrimidine at this position, as present in the UGUUC sequence from the loop region, could not form these interactions. NMR CSP and RNA unwinding experiments with different RNAs confirm that the loop sequence is not recognized by Hera_RBD. In the YxiN_RBD/RNA complex, the first uridine of the UGUUC sequence does not bind at all to the protein but is located approximately 15 Å away from the position that Gua1 adopts in the Hera_RBD complex. Gua3 in the Hera/RNA complex and the corresponding uridine of the YxiN/RNA complex are located in the same position. However, Gua3 does not engage in base-specific interactions with Hera_RBD, but forms a hydrophobic interaction with the side-chain of K455, and a salt bridge with the protonated amino group through its 5′-phosphate. In the YxiN RBD, the corresponding residue to K455 is an alanine, precluding formation of a salt bridge. Instead, YxiN residue K427 (corresponding to position G456 in Hera) hydrogen bonds to a uridine base (corresponding to Gua3 in Hera) and the 2′-hydroxyl group, and additionally forms a salt bridge with the 3′-phosphate group. A guanine, as present in the Hera/RBD complex, would form none of these three interactions, indicating that the RBDs dictate the recognition of different sequences at this position. Finally, the fourth nucleotide (Pyr4 in Hera, uridine in YxiN) packs against A452 and L453. The equivalent positions in YxiN are the smaller G423 and T424, and as a consequence, the uridine at this position in the RNA sequence is located approximately 3 Å closer to the YxiN RBD. The basic C-terminal sequence of Hera that contains five arginines and extends from the Hera_RBD binds to larger RNA substrates but has no counterpart in YxiN. Thus, the interaction of the RBDs of Hera and YxiN with RNA is substantially different.

The differences in interactions with Gua1 and Pyr4 in Hera and YxiN mark diversion points in the RNA traces of the Hera_RBD and YxiN_RBD complexes (Figure 9A). The resulting different paths of larger RNAs exiting the RBD will affect the RNA conformation further away from the central binding site, possibly enabling the interaction of the C-terminal tail with the adjacent hairpin. Furthermore, the structural differences of RNA bound at the RBD may have functional consequences for RNA presentation by the RBD to the helicase core for unwinding.

### Implications for Hera function

Unwinding of the 32/9mer RNA by Hera is not caused by binding of the RBD to the region adjacent to the duplex, but requires the helicase core. In contrast to YxiN (11,40), Hera does not specifically recognize the loop in hairpin 92 of 23S rRNA, but interacts with the adjacent single-stranded region, and the double-stranded stem. Possibly, the observed binding of Hera to RNase P RNA with a concomitant activation of the helicase core (7) also results from interactions with (abundant) GGGPyr sequences. Thus, Hera is most likely not specifically involved in ribosome biogenesis or RNase P maturation, but generally binds to exposed GGGPyr sequences flanked by double-stranded regions through its RBD. The helicase core then unwinds adjacent duplex regions. Such a mode of action would be reminiscent of Cyt-19 (41), a DEAD box protein that binds to structured regions and unwinds duplexes nearby (12,42). Cyt-19 is involved in splicing (41), but also acts as a general RNA chaperone (12). The natural Hera RNA substrates are currently unknown, and the *in vivo* function of Hera remains ill-defined. A function of Hera as a general RNA chaperone would predict that it acts on a variety of RNA substrates. Future mechanistic studies will therefore have to focus on the action of Hera on the yet-to-be-identified natural RNA substrates.

## ACCESSION NUMBERS

PDB-IDs 4I69, 4I68 and 4I67.

## SUPPLEMENTARY DATA

Supplementary Data are available at NAR Online: Supplementary Table 1 and Supplementary Figures 1–7.

Supplementary Data
