# Supplementary material for: Recognition of two distinct elements in the RNA substrate by the RNA-binding domain of the T. thermophilus DEAD box helicase Hera
Source: Nucleic Acids Res. 2013 Apr 25;41(12):6259–72. doi: 10.1093/nar/gkt323 (PMC3695512; doi:10.1093/nar/gkt323)
Supplement: Supplementary Data [file supp_41_12_6259__index.html]

Recognition of two distinct elements in the RNA substrate by the RNA-binding domain of the T. thermophilus DEAD box helicase Hera — Recognition of two distinct elements in the RNA substrate by the RNA-binding domain of the T. thermophilus DEAD box helicase Hera — Supplementary Data 

# Recognition of two distinct elements in the RNA substrate by the RNA-binding domain of the *T. thermophilus* DEAD box helicase Hera

## Supplementary Data

files

**Files in this Data Supplement:**

- Supplementary Data - pdf file
