# Supplementary material for: Recognition of two distinct elements in the RNA substrate by the RNA-binding domain of the T. thermophilus DEAD box helicase Hera
Source: Nucleic Acids Res. 2013 Apr 25;41(12):6259–72. doi: 10.1093/nar/gkt323 (PMC3695512; doi:10.1093/nar/gkt323)
Supplement: Supplementary Data [file supp_gkt323_nar-00593-r-2013-File011.pdf]

Supplementary information

Recognition of two distinct elements in the RNA substrate by the RNA binding domain of the *T. thermophilus* DEAD box helicase Hera

Lenz Steimer[1,#], Jan Phillip Wurm[2,#], Martin H. Linden[3,+], Markus G. Rudolph[4], Jens Wöhnert[2,*] and Dagmar Klostermeier[1,*]

1 University of Muenster, Institute for Physical Chemistry, Corrensstrasse 30, D-48149 Muenster, Germany, Phone: 0049-251-83 23421, FAX 0049-251-83 29138, dagmar.klostermeier@uni-muenster.de

2 University of Frankfurt, Institute for Molecular Biosciences, Center for Biomolecular Magnetic Resonance (BMRZ), Max-von-Laue-Str.9, D-60438 Frankfurt/M., Phone: 0049-69-798 29271, FAX 0049-69-798 29527, woehnert@bio.uni-frankfurt.de

3 University of Basel, Biozentrum, Klingelbergstrasse 70, CH-4056 Basel, Switzerland

4 F. Hoffmann-La Roche AG, pRED, Pharma Research & Early Development, Discovery Technologies, Grenzacherstrasse 124, CH-4070 Basel, Switzerland

* corresponding authors

# equal contributions

+ present address: German Institute of Food Technologies, Prof.-von-Klitzing-Str. 7, D-49610 Quakenbrück, Germany

Suppl. Figure 1: **Time-dependent changes in anisotropy upon binding of RNA to Hera**

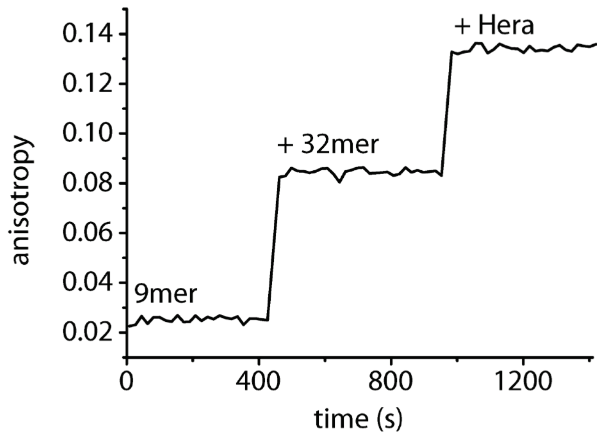

A 3'-fluorescein-labeled 9mer RNA (500 nM) exhibits a low fluorescence anisotropy of r = 0.025 due to rapid rotational motion. The anisotropy increases to r = 0.085 when addition of 1 µM 32mer RNA allows for duplex formation, leading to slower rotational tumbling. Addition of 1 µM Hera and formation of the Hera-32/9mer complex caused an instantaneous increase in anisotropy to r = 0.135, justifying a 2 min equilibration time in anisotropy titrations. Measurements were performed in 50 mM Tris pH 7.5, 150 mM NaCl, 5 mM $MgCl_2$ at 25°C. Fluorescence was excited at 495 nm and detected at 530 nm.

Suppl. Figure 2: **Binding of RNA to Hera and Hera domains**

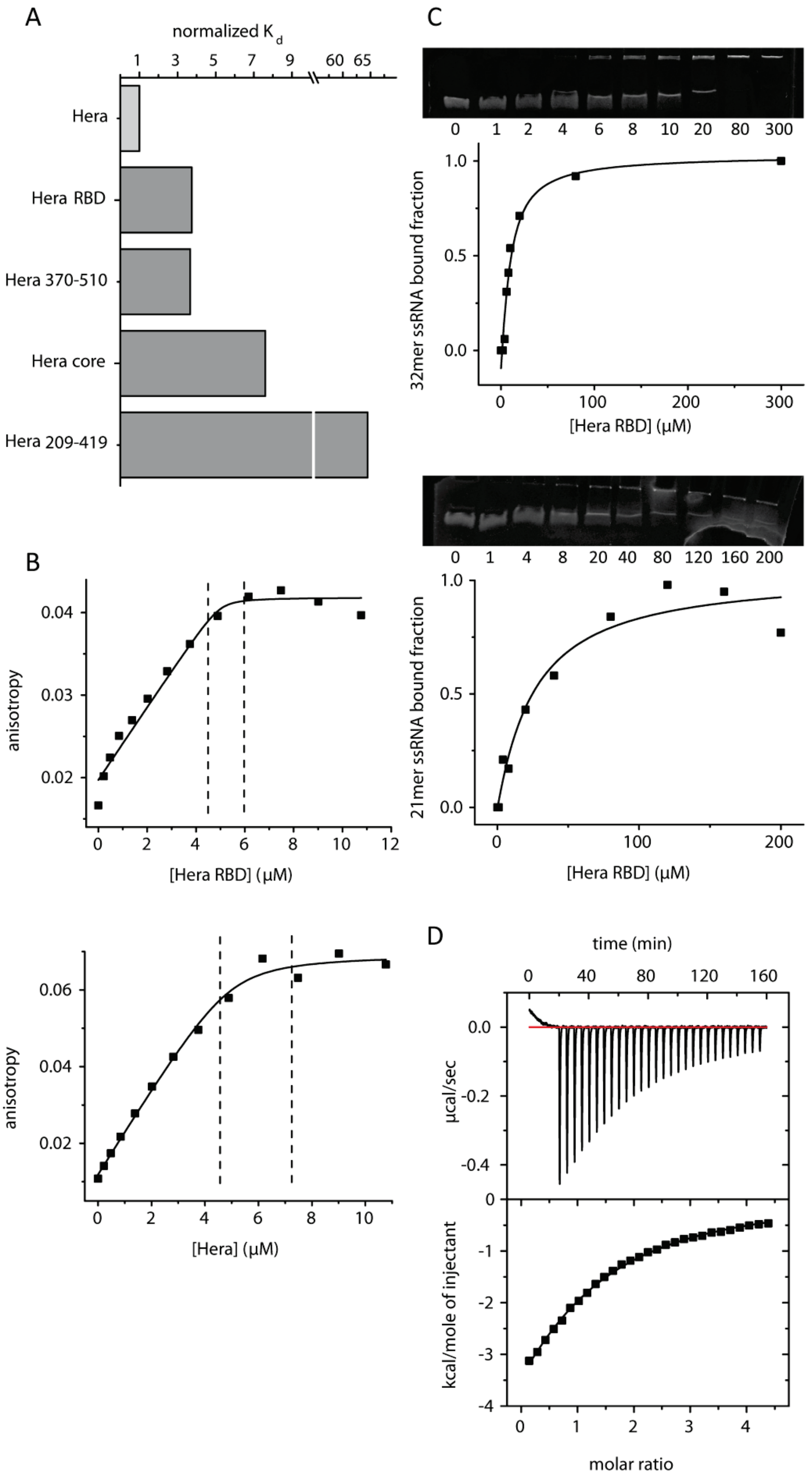

(A) Comparison of K_d values of 32mer RNA complexes of Hera_RBD, Hera_370-510 (DD, RBD), Hera_core and Hera_208-419 (RecA_C, DD) with full-length Hera. The values are consistent with the RBD as the major RNA binding platform in the absence of ATP. The core contributes to RNA binding, but the DD does not. Measurements were performed in 50 mM Tris pH 7.5, 150 mM NaCl, 5 mM $MgCl_2$ at 25°C with 50 nM 5´-fluorescein-labeled 32mer RNA. Fluorescence was excited at 495 nm and detected at 530 nm.
(B) Stoichiometric titrations of the 5´-fluorescein-labeled 32mer (5 μM) with Hera and Hera_RBD. The broken lines indicate the lower and upper limit of the equivalence points. The data support a 1:1 stoichiometry of RNA binding to the RBD and to Hera (one RNA molecule per Hera protomer). Measurements were performed in 50 mM Tris pH 7.5, 150 mM NaCl, 5 mM $MgCl_2$ at 25°C. Fluorescence was excited at 495 nm and detected at 530 nm.
(C) Electrophoretic mobility shift assay of 32mer and 21mer binding to Hera_RBD, and quantification of the RNA fraction bound. Samples were incubated in 50 mM Tris/HCl, pH 7.5, 150 mM NaCl and 12% (v/v) glycerol at 25°C for 30 min prior to gel electrophoresis. The numbers below the lanes indicate the protein concentration in µM. The RNA concentration was 6 µM. The K_d values can be estimated to ~7 µM (32mer) and ~23 µM (21mer).
(D) Isothermal calorimetric titration of the 21mer RNA with Hera in 50 mM Tris pH 7.5, 150 mM NaCl, 5 mM $MgCl_2$ at 25 °C. 20 µM 21mer RNA in the cell was titrated with Hera_RBD (400 µM). The injection time was 20 s, with 300 s equilibration time between injections. The lack of an initial baseline precludes quantification of the K_d value, but it can be estimated to ~ 40 µM, consistent with the value determined by EMSA (panel C) and NMR (see main text).

Suppl. Figure 3: **Complete assignment of ^{1}H,^{15}N-HSQC spectrum, and changes in chemical shifts upon binding of different RNAs**.

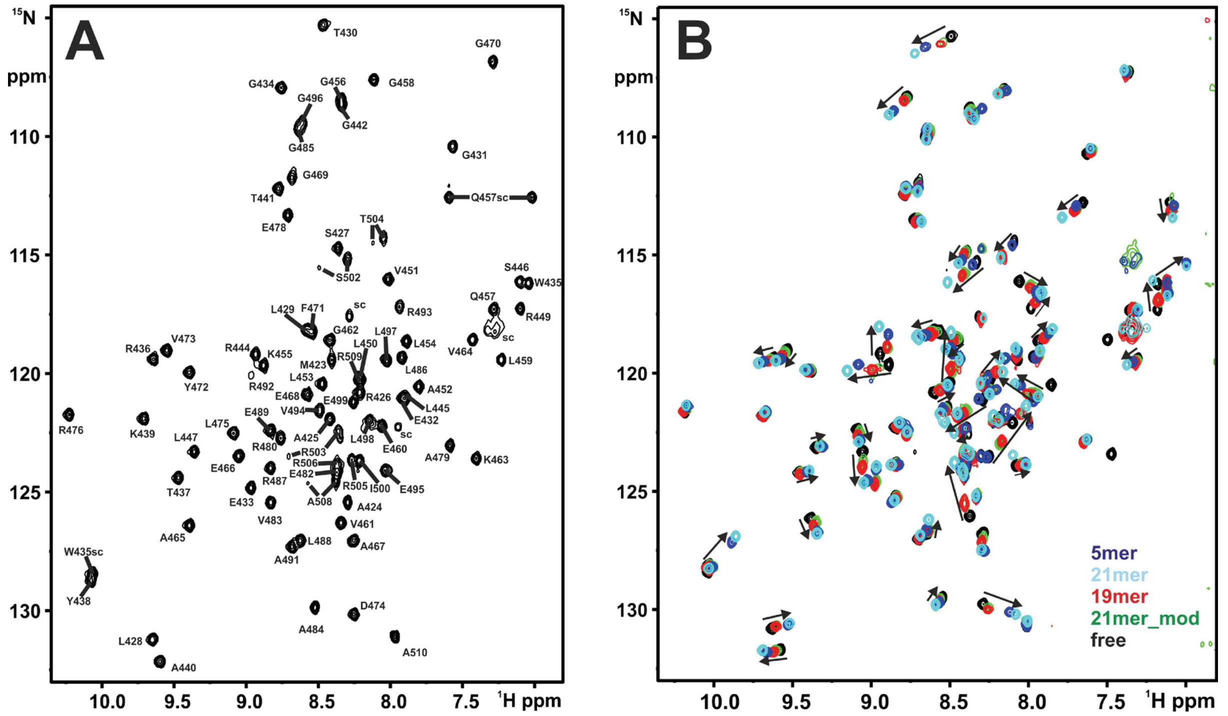

(A) Complete assignments for the backbone amide groups of the Hera-RBD in its free form. Shown is the ^{1}H,^{15}N-HSQC spectrum recorded at 37°C with all assignments indicated. The two signals for the single side chain amide group of Q457 are connected by a horizontal line and labeled as Q457sc. The signal of the side chain amide group of W435 is labeled as W435sc. Unassigned arginine side chain signals which are folded into the spectrum are labeled as sc.

(B) Overlay of the ^{1}H,^{15}N-HSQC spectra of the free RBD (black) and spectra in the presence of 5mer (dark blue), 19mer (red), 21mer (light blue), and 21mer_mod RNA (green). The directions of the chemical shift changes are indicated by the arrows. Spectra were measured in 25 mM Bis-Tris/HCl, pH 6.0, 50 mM NaCl, 10% (v/v) D_2O at 37°C. The Hera_RBD concentration was 100 μM.

Suppl. Figure 4: **NMR and CD analysis of RBD secondary structure**

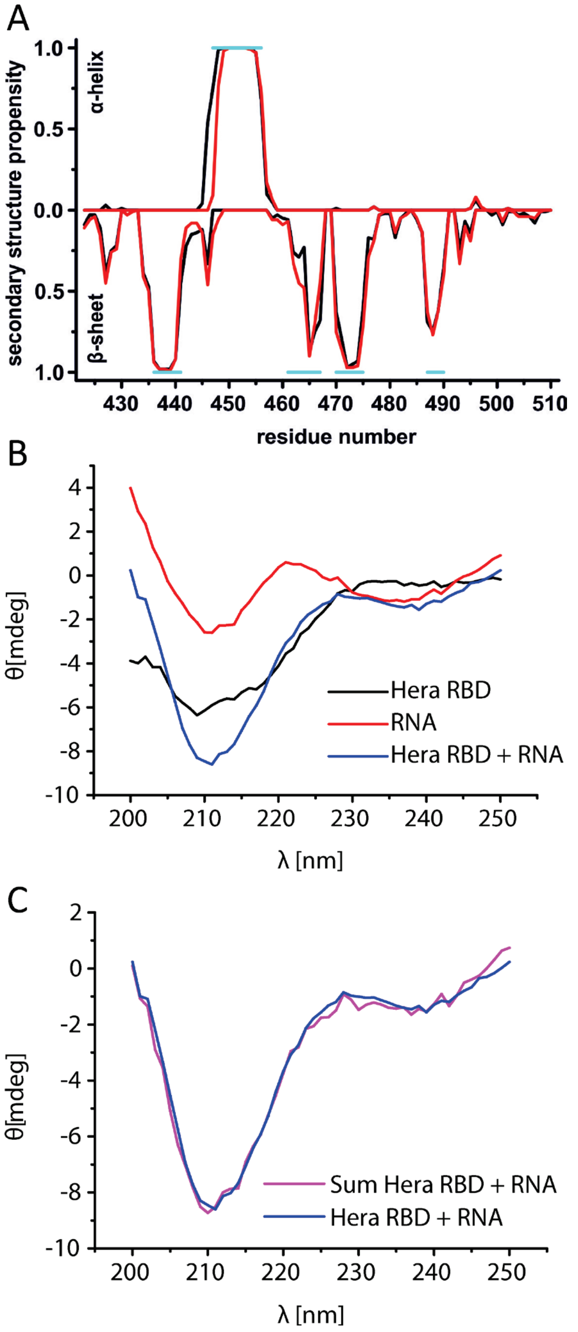

(A) Influence of 21mer RNA binding on secondary structure propensities in the Hera-RBD. Shown are the results of a PECAN-analysis of the backbone chemical shifts (H_N,N^H, CO, CA, CB) for the free protein (black) and protein bound to the 21mer RNA (red). Light blue lines indicate the positions of secondary structure elements as observed in the X-ray structure. Spectra were measured in 25 mM Bis-Tris/HCl, pH 6.0, 50 mM NaCl, 10% (v/v) D_2O at 37°C.

(B) Far-UV CD spectra of Hera_RBD (black), 32mer RNA (red), and the RBD/32mer RNA complex (blue). Spectra were measured with 10 μM protein in 50 mM potassium phosphate buffer, pH 7.5 at 25°C.

(C) The spectrum of the RBD/RNA complex (blue) is identical to the sum of the individual spectra (purple), indicating that RNA binding does not induce secondary structure formation.

Suppl. Figure 5 : **The affinity of Hera_RBD for the GGGC-RNA is independent of the C-tail**

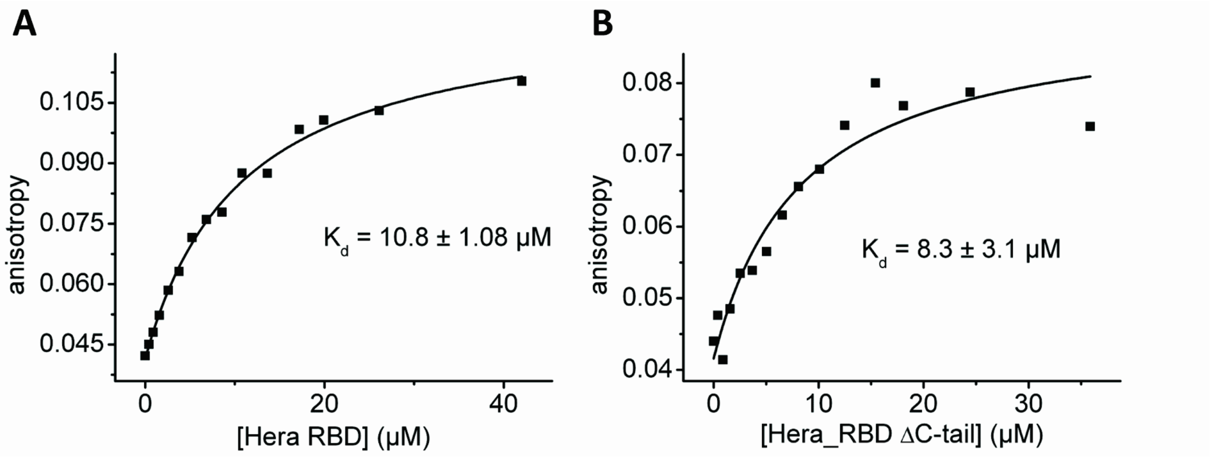

Fluorescence anisotropy titration of a 5'-fluorescein-labeled GGGC-RNA with (A) Hera_RBD and (B) Hera_RBD_ΔC-tail. The K_d values for the RNA/RBD complexes are 8 µM in the absence and 11 µM in the presence of the C-tail, indicating that the C-tail does not contribute to binding of this RNA. The K_d value for the RBD/GGGC complexes are virtually identical to the K_d value of the Hera_RBD_ΔC-tail/32mer complex (13 µM, cp. Figure 2, main text). These data are in-line with the core of Hera_RNA binding the GGGU sequence in the single-stranded region of the 32mer RNA, and the ΔC-tail contacting the hairpin. Measurements were performed with 50 nM RNA in 50 mM Tris/HCl, pH 7.5, 150 mM NaCl with GST-Hera_RBD at 25°C. Fluorescence was excited at 495 nm and detected at 530 nm.

Suppl. Figure 6: **Structural features of RBD-bound RNA**

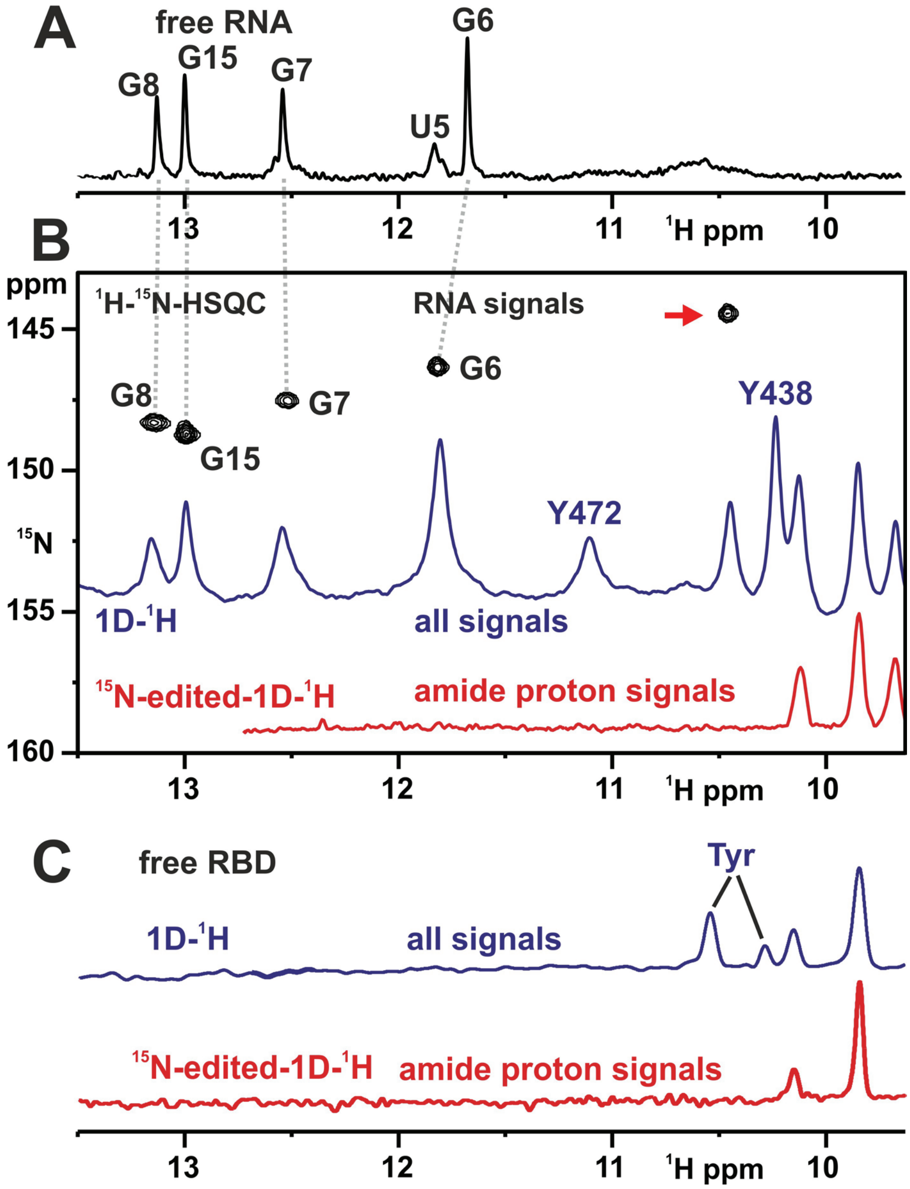

(A) Imino proton region of a 1D-^{1}H-spectrum of the free 21mer RNA. 5 signals with chemical shifts typical for imino protons of Watson-Crick base pairs are visible and their NOE-based assignment is given. (B) Overlay of the imino group region of the ^{1}H,^{15}N-HSQC of ^{15}N-guanine-labelled 21mer RNA bound to unlabelled Hera-RBD with a ^{15}N-decoupled 1D-^{1}H-NMR-spectrum of the RNA/protein-complex and a ^{15}N-selected-1D-1H-NMR-spectrum of 21mer RNA bound to ^{15}N-labelled Hera-RDB

(B) Comparison of the three spectra shows that of the 10 signals observed in this spectral range three belong to protein backbone amide groups (red spectrum). Two are not correlated to ^{15}N-nuclei when either ^{15}N-labelled RNA or ^{15}N-labelled protein is used and therefore most likely belong to protein OH-groups. NOESY-spectra suggest that these signals belong to two protein tyrosine residues. Five signals represent guanine imino groups of the bound RNA. Four of these signals have chemical shifts typical of guanine imino groups in Watson-Crick-base pairs and belong to the helical stem of the 21mer RNA which is still present when the RBD is bound. The fifth signal (red arrow) belongs to a hydrogen-bonded

guanine not in a canonical base pairing interactions. Moreover, this signal is not observable in the free RNA (not shown). Thus, this signal corresponds most likely to a guanine interacting directly with the protein via hydrogen bonding in agreement with the binding mode for Gua2 in the X-ray structure of the Hera-RBD in complex with 5'-GGGC-3'.
(C) Comparison of 1D- and ^{15}N-filtered 1D-spectra of the free RBD show that the two signals belonging to OH-groups are already observable in the free protein and that their protection from exchange is not caused by RNA-binding. All spectra were measured in 25 mM Bis-Tris/HCl, pH 6.0, 50 mM NaCl, 10% (v/v) D_2O at 37°C.

Suppl. Figure 7: **RNA unwinding by Hera**

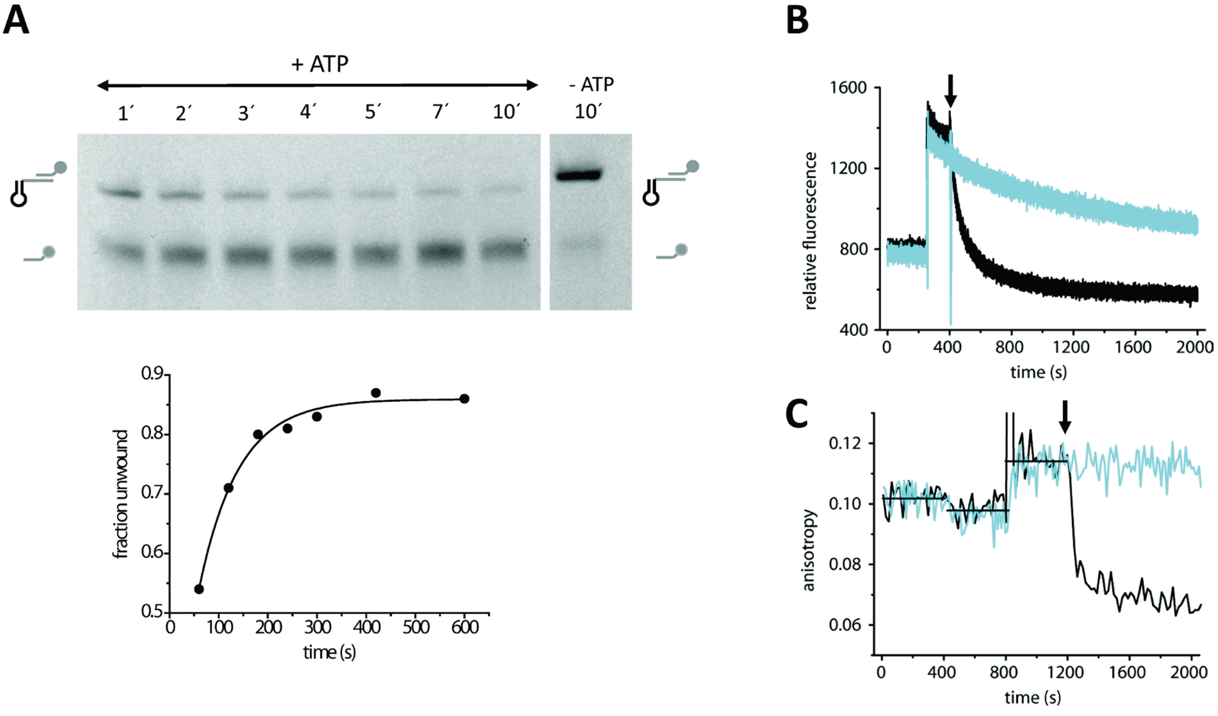

(A) Unwinding of the 32/9mer RNA substrate (0.5 μM, containing a 3'-fluorescein-9mer) by Hera (5 μM) in the presence of 5 mM ATP. The unwinding reaction was stopped at different time-points, and double-stranded substrate and single-strand released were separated by native PAGE. Quantification of the fraction of RNA unwound (bottom panel) reveals that Hera unwinds the 32/9mer RNA substrate with a rate constant of 0.012 s^{-1}.

(B) Unwinding of a donor/acceptor-(Cy3/Cy5)-labeled 32/9mer RNA substrate (0.5 μM), followed by a reduction in acceptor fluorescence due to a loss of FRET upon release of the single strand. Cy3 fluorescence was excited at 554 nm and Cy5 fluorescence detected at 666 nm. Hera (5 μM) unwinds the 32/9mer RNA substrate with a rate constant of 0.017 s^{-1} (plus a slower phase with k = 0.003 s^{-1}) in the presence of 1 mM ATP (black, arrow). No unwinding (above background) is detected in the presence of 1 mM ADPNP (cyan).

(C) Unwinding of the 32/9mer RNA substrate (50 nM 9mer, 1 μM 32mer) followed by fluorescence anisotropy of a fluorescein attached to the 3´-end of the 9mer. Fluorescence was excited at 495 nm and detected at 530 nm. In the 32/9mer RNA the anisotropy is 0.103. Addition of 500 nM trap RNA (unlabeled 9mer) leads to a slight decrease in anisotropy. Formation of the Hera-32/9mer -complex results in an anisotropy of r = 0.115. After ATP addition (4 mM, arrow), the anisotropy decreases due to release of the 9mer upon duplex unwinding. ADPNP (4 mM) does not support unwinding (cyan). The half-life of the unwinding reaction is ca. 45 s, corresponding to a rate constant of unwinding of ca. 0.015 s^{-1}.

All reactions were performed in 50 mM Tris pH 7.5, 150 mM NaCl, 5 mM $MgCl_2$ at 25°C in the presence of a 10-fold excess of non-labeled trap strand to ensure single turnover conditions. The similar rate constants of unwinding and the lack of unwinding in the absence of ATP (not shown) and in the presence of ADPNP in all three assays confirm that these methods report on duplex unwinding.

Supplementary Table 1: **Hera_RBD/RNA interactions.**

RNA	Hera_RBD	Dist. (Å), or #	Type
Gua1 base	Lys463 side-chain		vdW
Gua1 base	Gly462 amide		vdW
Gua1 N1	Glu432 Oε2	3.0	H-bond
Gua1 N2	Glu432 Oε2	3.3	H-bond
Gua1 O6	Thr430 Oγ1	3.5	Weak H-bond
Gua2 base	Leu447		vdW
Gua2 ribose	Val451		vdW
Gua2 OP2	Lys455 Nζ	3.5	Charged H-bond
Gua2 N1	Val461 O	3.1	H-bond
Gua2 N1	Gly462 O	2.8	H-bond
Gua2 N2	Gly462 O	2.8	H-bond
Gua2 O6	Lys463 N	2.8	Weak H-bond
Gua3 base	Lys455 side-chain		vdW
Gua3 base	Gly456		vdW
Gua3 ribose	Pro448		vdW
Gua3 ribose	Ala452		vdW
Pyr4 base	Ala452		vdW
Pyr4 base	Leu453		vdW
Pyr4 base	Arg449 side-chain		vdW
Pyr4 O2	Arg449 Nη1	2.7	H-bond

H-bond: hydrogen bond, vdW: van der Waals interactions. Only significant vdW interactions are listed.
